# Blockade of the estrogen receptor alpha–pregnane X receptor axis protects ovariectomized mice against ethanol-induced hepatotoxicity

**DOI:** 10.1016/j.jbc.2025.110238

**Published:** 2025-05-15

**Authors:** Elizabeth Twum, Malvin Ofosu-Boateng, Daniel O. Nnamani, Lidya H. Gebreyesus, Nour Yadak, Kusum K. Kharbanda, Frank J. Gonzalez, Maxwell A. Gyamfi

**Affiliations:** 1 Department of Pharmaceutical Sciences, The University of Tennessee Health Science Center, Memphis, Tennessee, USA; 2 Department of Pathology and Laboratory Medicine, The University of Tennessee Health Science Center, Memphis, Tennessee, USA; 3 Research Service, Veterans Affairs Nebraska-Western Iowa Health Care System, Omaha, Nebraska, USA; 4 Department of Internal Medicine, University of Nebraska Medical Center, Omaha, Nebraska, USA; 5 Department of Biochemistry & Molecular Biology, University of Nebraska Medical Center, Omaha, Nebraska, USA; 6 Center for Cancer Research, National Cancer Institute, Bethesda, Maryland, USA

**Keywords:** alcohol-associated fatty liver disease, females, ovariectomy, oxidative stress, pregnane X receptor

## Abstract

Women develop alcohol-associated liver disease (ALD) faster than men at any level of alcohol consumption, implicating estrogen as a contributing factor. However, the precise mechanism remains unknown. Therefore, 12-week-old female C57BL/6N mice were subjected to either bilateral ovariectomy (OVX) or sham surgery. After a 3-week recovery period, the mice were fed either a 5% ethanol (EtOH)-containing liquid diet or paired-fed control diet for 10 days followed by a single gavage dose of EtOH (5 g/kg, 30% EtOH solution). The mice were examined for serum biochemical parameters, hepatotoxicity, histology, expression of xenobiotic nuclear receptors, pregnane X receptor (PXR) and constitutive androstane receptor, and their target gene mRNAs and proteins in hepatic and perigonadal white adipose tissues (pgWATs). While OVX mice on a control diet significantly gained weight, EtOH significantly increased hepatotoxicity, residual EtOH levels, lipid peroxidation, and oxidative stress in sham-operated mice but not in their OVX counterparts. In addition, in the livers and pgWAT of the sham mice, EtOH significantly increased the mRNA and/or protein levels of the major estrogen receptor (estrogen receptor α), PXR, constitutive androstane receptor, and their target genes, proinflammatory cytokines and chemokines, lipogenic genes, and fibroblast growth factor 21 levels, a predictive biomarker for ALD severity in humans, but inhibited nuclear factor erythroid 2-related factor 2 (NRF2) and its target genes encoding NQO1 and BHMT (betaine-homocysteine *S*-methyltransferase). Unexpectedly, all these changes were attenuated in the EtOH-fed OVX mice by the upregulation of NRF2 and aryl hydrocarbon receptor and their downstream antioxidant target genes. Together, these results suggest the existence of an estrogen-regulated estrogen receptor α–PXR–NRF2 signaling axis in liver and pgWAT, which contributes to sexual dimorphism in ALD.

Alcohol-associated liver disease (ALD) involves liver tissue damage because of excessive alcohol intake, either episodically or chronically, leading to fat accumulation, inflammation, fibrosis, and cirrhosis (1). Diagnosis of ALD requires a history of sustained heavy drinking and the exclusion of autoimmune hepatitis, drug-induced liver disorders, and other non–alcohol-related liver diseases (2). Key risk factors include drinking patterns, ethnicity, female sex, adolescence, poor nutrition, obesity, hepatitis C infection, and genetic polymorphisms in genes, such as cytochrome P450 2E1 (CYP2E1), glutathione-*S*-transferases (GSTs), tumor necrosis factor-alpha (TNF-α), and aldehyde dehydrogenase 2 (ALDH2) (3, 4). The initial stage of ALD is hepatic steatosis, which is characterized by fat buildup in liver cells because of disrupted lipid metabolism, increased insulin resistance, and inflammation (5). ALD can progress to more severe forms, including steatohepatitis, cirrhosis, and hepatocellular carcinoma, which contribute to 30% to 50% of cirrhosis-related deaths globally (6, 7). In steatosis, fat, primarily triglycerides, accumulates to exceed 5% to 10% of liver weight (8). Alcohol impairs lipid metabolism, leading to fat buildup through mitochondrial β-oxidation suppression and increased fatty acid synthesis (3).

The World Health Organization estimated that alcohol consumption contributes to 5.1% of the global disease burden, with alcohol-related causes responsible for 3 million deaths and 22.2 million disability-adjusted life years worldwide, including 607,000 deaths because of alcohol-induced liver cirrhosis (9). In 2022, the Centers for Disease Control and Prevention reported a 23% increase in alcohol-related deaths in the United States, with ALD as the leading cause (10). Alarmingly, alcoholic cirrhosis deaths have risen significantly among those younger than 45 years, especially women aged 25 to 34 (11). In addition, a study comparing US alcohol use patterns from 2001–2002 to 2012–2013 showed an 80% rise in heavy drinking among women and a 30% rise among men (12).

While alcohol consumption is generally more common among men, women tend to experience more severe adverse effects (13). From 2009 to 2015, women had a higher incidence of alcohol-related liver disease at 50% compared with 30% in men (14, 15). Several factors contribute to this increased susceptibility in women, including higher blood alcohol concentrations (BACs) because of lower body water content, which increases the absorption of alcohol for a given intake (16). Women also have lower stomach alcohol dehydrogenase (ADH) activity, which reduces first-pass ethanol (EtOH) metabolism, exposing the liver to higher EtOH levels as more alcohol enters portal circulation. This sex difference in ALD risk is further attributed to sex-based responses in liver-resident Kupffer cells, which show heightened sensitivity to endotoxins in females, a condition exacerbated by alcohol-induced gut permeability that allows endotoxins and microbial antigens to reach the liver, intensifying inflammation, evident in studies showing higher plasma endotoxin levels in women than men after binge drinking (17, 18).

In addition, estrogen and growth hormone in women modulate hepatic enzymes such as CYP2E1, involved in EtOH metabolism, leading to elevated oxidative stress and liver injury because of mitochondrial DNA damage, lipid peroxidation (LPO), and increased cytokine production (19). Estrogen, specifically, has been implicated in enhancing liver inflammation, oxidative stress, and Kupffer cell sensitivity to endotoxins, thus contributing to heightened female vulnerability to ALD (18). Notably, studies have shown that estrogen administration reverses the protective effects of ovariectomy (OVX) against EtOH-induced hepatotoxicity, further emphasizing its role in ALD pathogenesis (20). Understanding the role of estrogen in ALD sexual dimorphism is further complicated by reports indicating that exogenous estrogen administration protects against hepatic lipid accumulation and hepatotoxicity induced by both obesity or high-fat diet and alcohol consumption (20, 21, 22). Importantly, the specific cellular mechanisms by which estrogen controls metabolic homeostasis and sex-specific susceptibility to EtOH-induced hepatotoxicity are not completely understood. Moreover, currently, no Food and Drug Administration–approved treatments exist for ALD at any stage, highlighting the need to better understand ALD pathophysiology to identify therapeutic targets, especially for women.

The goal of this study was to investigate whether the reduction in blood estrogen levels by OVX would decrease the sensitivity of female mice to EtOH-induced liver injury and to determine the mechanisms involved. Therefore, 12-week-old female C57BL/6N mice were subjected to either bilateral OVX to remove the major site of endogenous estrogen synthesis or sham surgery. Mice were allowed to recover for 3 weeks, divided into four groups, and paired-fed either a control diet or 5% EtOH-containing diet for 10 days followed by a single oral dose of EtOH (5 g/kg, 30% EtOH solution). Here, we showed that female sham mice are at a greater risk of EtOH-induced liver injury compared with OVX females. We also established that the protective effect observed in the OVX females was mediated by the estrogen receptor α (ERα)–pregnane X receptor (PXR)–nuclear factor erythroid 2-related factor 2 (NRF2) and aryl hydrocarbon receptor (AhR) signaling pathways, resulting in an overall decrease in oxidative stress and inflammation in the OVX EtOH-fed mice.

## Results

### OVX-induced weight gain occurs independent of food consumption

**Figure 1:**
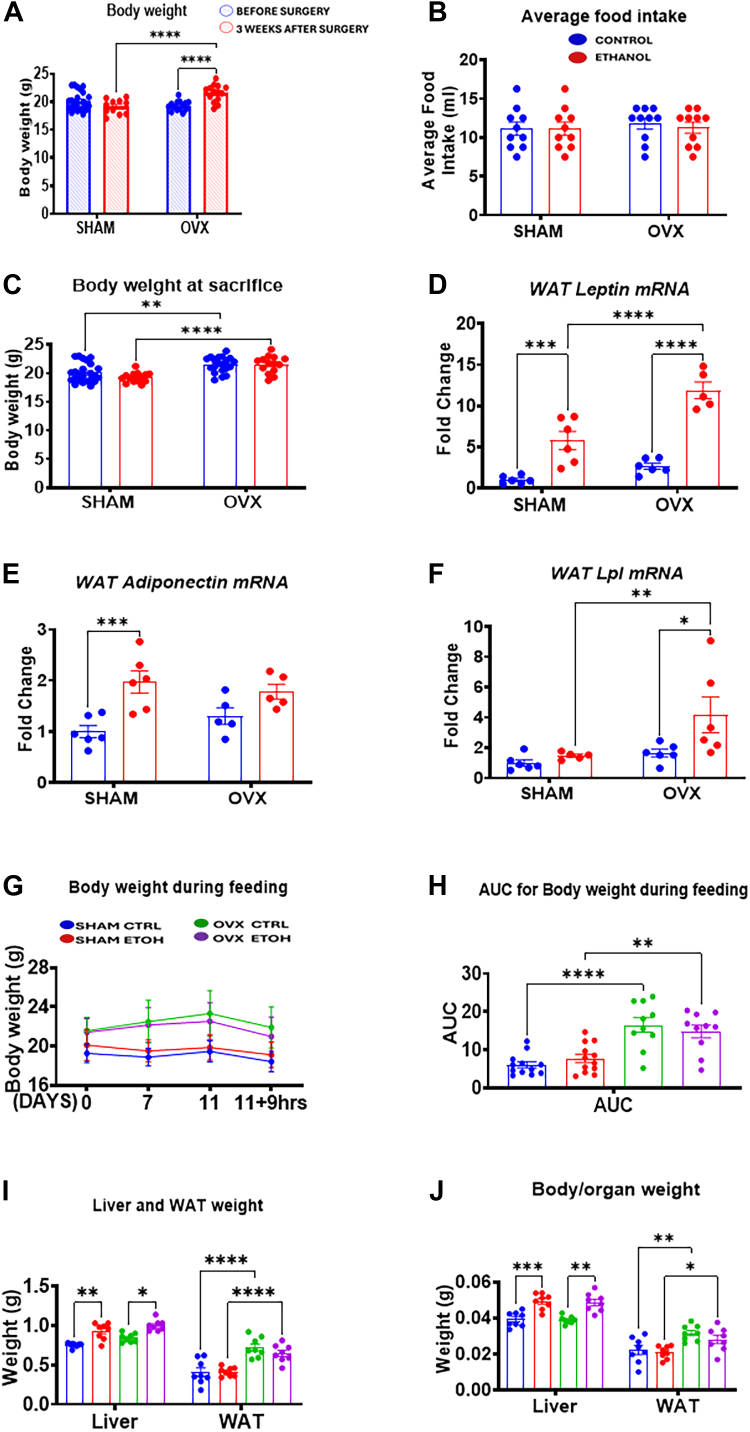
**Effect of sham and ovariectomy (OVX) surgeries on body weight changes, organ weight, food intake, WAT lipoprotein lipase, and adipokine gene expression levels.** Female C57BL/6N (WT) mice were subjected to either bilateral OVX or sham surgery. After a 3-week recovery period, the mice were pair-fed either a 5% ethanol (EtOH)-containing liquid diet or control diet for 10 days followed by a single oral dose of EtOH (5 g/kg, 30% EtOH solution) on day 11, and the mice were anesthetized with isoflurane and tissues were collected. Total RNA was extracted from perigonadal white adipose tissues (pgWATs), and complementary DNA was prepared for mRNA quantification with *Gapdh* mRNA as the house-keeping gene described in the Experimental procedures section. *A*, body weight before surgery and 3 weeks postsurgery, *B,* average food intake per mouse, *C*, body weight at sacrifice, *D*, pgWAT leptin (*Lep*) mRNA, *E*, pgWAT adiponectin (*Adipoq*) mRNA, *F*, pgWAT lipoprotein lipase (*Lpl*) mRNA, *G*, body weight changes during the feeding period until euthanasia, *H*, area under the curve for body weight changes during the feeding period, *I*, liver and pgWAT weight at sacrifice, *J*, liver-to-body weight ratio and WAT-to-body weight ratio. Data are presented as mean ± SEM (n = 6–8). ∗*p* < 0.05, ∗∗*p* < 0.01, ∗∗∗*p* < 0.001, and ∗∗∗∗*p* < 0.0001 between indicated groups. WAT, white adipose tissue.

Presurgery analysis showed no differences in body weight among the groups. However, 3 weeks postsurgery, OVX mice exhibited a significant body weight increase compared with sham mice (Fig. 1*A*), which persisted throughout the study (Fig. 1, *C*, *G*, and *H*). During the chronic-binge alcohol feeding, all groups consumed similar diet volumes (Fig. 1*B*); however, both control- and EtOH-fed OVX mice displayed notably higher body weights at the study's end compared with their respective sham groups (Fig. 1*C*). In agreement, both the control and EtOH-fed OVX groups showed significant increases in perigonadal white adipose tissues (pgWATs) and white adipose tissue-to-body weight ratio compared with sham counterparts (Fig. 1, *I* and *J*). Liver weight and liver-to-body weight ratios were also significantly elevated in EtOH-fed groups compared with their controls (Fig. 1, *I* and *J*). To explore the cause of weight gain in OVX groups, we analyzed mRNA levels of lipoprotein lipase (Lpl), leptin, and adiponectin in pgWAT (Fig. 1, *D*–*F*). Lpl, crucial in lipid metabolism, facilitates nonesterified fatty acid (NEFA) storage in adipose tissue, with increased LPL activity linked to greater fat storage and weight gain. Hormonal regulation of Lpl expression includes estrogen, which suppresses Lpl mRNA in adipose cells (23, 24). We observed a significant Lpl mRNA increase only in EtOH-fed OVX mice compared with all other groups (Fig. 1*F*). Leptin mRNA levels showed a similar trend, with the highest levels in EtOH-fed OVX mice than the increase we observed in EtOH-fed sham mice (Fig. 1*D*). In contrast, adiponectin mRNA levels were significantly elevated only in EtOH-fed sham mice (∼2.0-fold) compared with their control group (Fig. 1*E*). These findings suggest that OVX promotes weight gain independently of diet.

### Estrogen deficiency suppresses liver injury in female mice

**Figure 2:**
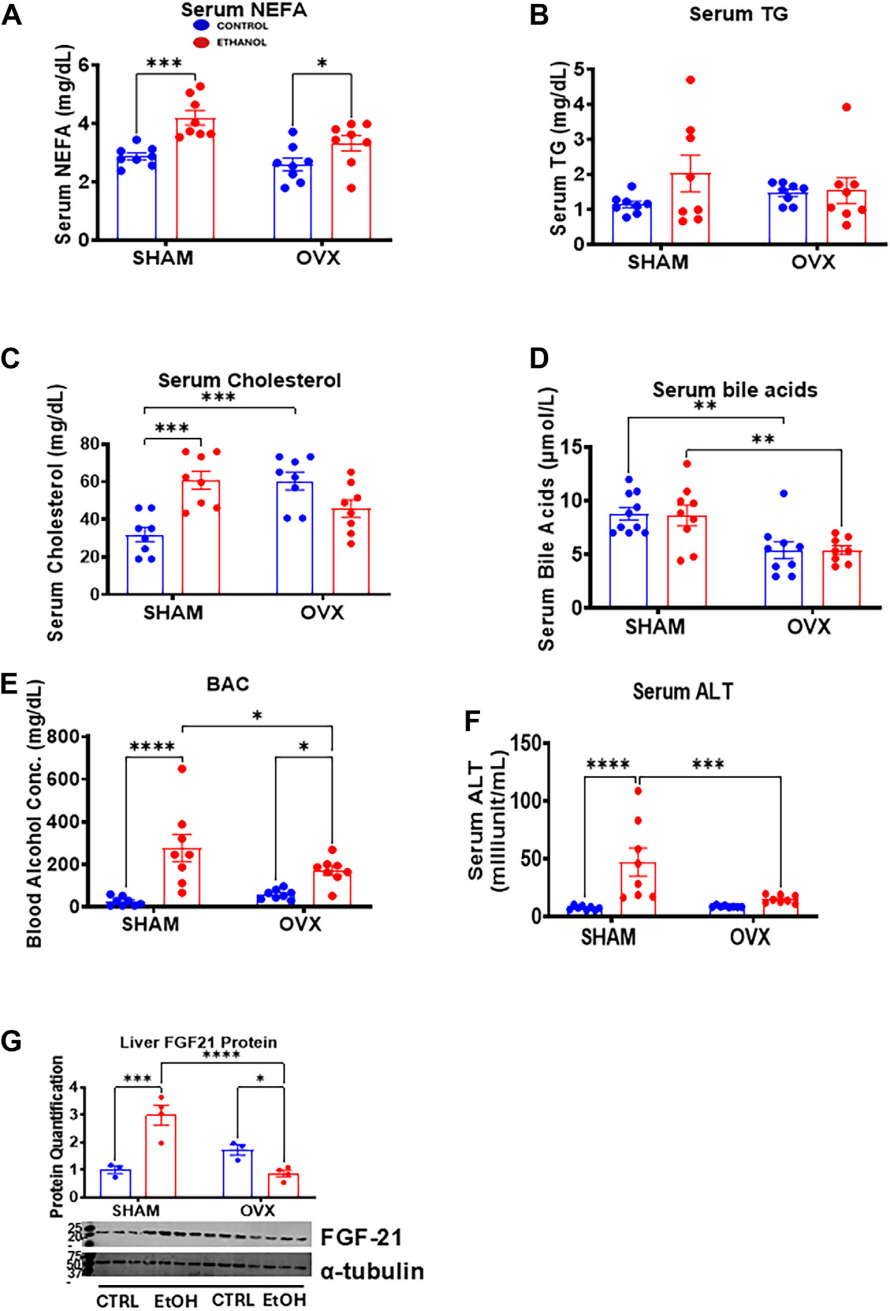
**Effect of sham and ovariectomy (OVX) surgeries on serum lipids, bile acids, blood alcohol concentration (BAC), alanine aminotransferase (ALT), and FGF21 protein levels.** Female C57BL/6N (WT) mice were subjected to either bilateral OVX or sham surgery. After recovery, the sham and OVX mice were pair-fed either a 5% ethanol (EtOH)-containing liquid diet or control diet for 10 days followed by a single oral dose of EtOH (5 g/kg, 30% EtOH solution), and serum was extracted for the various assays, liver homogenate were prepared for the assays as described in the Experimental procedures section. *A*, Serum nonesterified fatty acid levels, *B*, serum triglyceride levels, *C*, serum cholesterol levels, *D*, serum bile acid levels BAC or residual alcohol concentration after sacrifice, *E*, BAC or residual alcohol concentration, *F*, serum ALT levels, *G*, hepatic FGF21 protein. Data are presented as mean ± SEM (n = 3–8). ∗*p* < 0.05, ∗∗*p* < 0.01, ∗∗∗*p* < 0.001, and ∗∗∗∗*p* < 0.0001 between indicated groups. FGF21, fibroblast growth factor 21.

Alcohol-induced hepatic steatosis characterized by accumulation of fats in the liver is associated with increases in circulating levels of NEFA (25). In agreement, we observed a significant increase in serum NEFA levels in both EtOH-fed sham mice (1.5-fold) and EtOH-fed OVX mice (1.3-fold) (Fig. 2*A*). There was no change in serum triglyceride levels between the groups after EtOH ingestion (Fig. 2*B*), but higher serum cholesterol levels were observed in control-fed OVX mice (1.9-fold) compared with the sham-control group (Fig. 2*C*). However, EtOH significantly increased serum cholesterol levels in the sham mice (1.9-fold) but not in the EtOH-fed OVX mice (Fig. 2*C*). While the basal serum bile acid levels were significantly higher in the sham groups compared with the OVX groups, EtOH did not have any significant effect on the bile acid levels (Fig. 2*D*). Unexpectedly, the EtOH-fed sham mice had a significantly higher BAC (10.6-fold) after the mice were euthanized compared with sham controls. In contrast, the BAC was increased only 3.0-fold in the EtOH-fed OVX mice (Fig. 2*E*). Consistent with this observation, serum alanine aminotransferase (ALT), a marker of liver injury, showed a significant increase only in the EtOH-fed sham mice (6.2-fold) compared with its pair-fed control and significantly higher than in EtOH-fed OVX mice (Fig. 2*F*) despite the consumption of similar volumes of EtOH diet (Fig. 1*B*) in both EtOH-fed groups. Fibroblast growth factor 21 (FGF21) is a mediator of liver metabolism and a marker of mitochondrial dysfunction and cirrhosis (26); therefore, we examined the FGF21 protein levels (Fig. 2*G*). Unexpectedly, the FGF21 protein significantly increased (3.0-fold) only in the EtOH-fed sham mice, which was significantly higher than in the EtOH-fed OVX mice (Fig. 2*G*). In contrast, the FGF21 protein levels significantly decreased in the EtOH-fed OVX mice by 50% compared with paired-fed OVX controls (Fig. 2*G*).

### Histology scores and hepatic lipid levels together with microsomal triglyceride transfer protein expression in EtOH-fed sham and OVX mice

**Figure 3:**
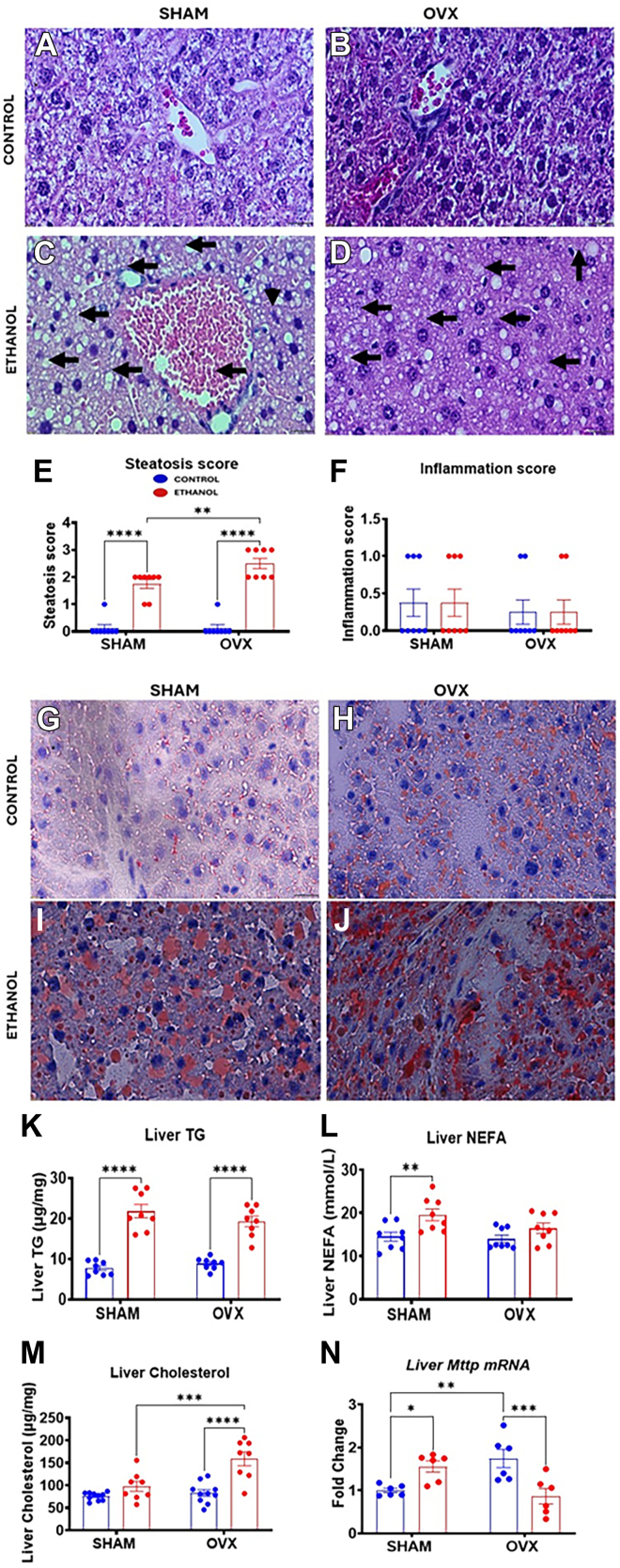
**Characterization of hepatic histology, hepatic lipids, and hepatic *Mttp* mRNA in female sham and ovariectomy (OVX) mice fed either control or ethanol (EtOH)-containing diets.** Female C57BL/6N (WT) mice were subjected to either bilateral OVX or sham surgery. After recovery, the sham and OVX mice were pair-fed either a 5% EtOH-containing liquid diet or control diet for 10 days followed by a single oral dose of EtOH (5 g/kg, 30% EtOH solution), and tissues were prepared for H&E or Oil-Red O staining. Besides, lipids were extracted for various assays, whereas mRNA was prepared for the assays as described in the Experimental procedures section. H&E staining of the liver for various treatment groups (original magnification 60×): *A*, pair-fed control sham, *B*, pair-fed control OVX, *C*, EtOH-fed sham, *D*, EtOH-fed OVX, *E*, Hepatic Steatosis Score, *F*, Hepatic Inflammation Score. Oil Red-O staining of liver tissue for various treatment groups (original magnification 60×), *G*, pair-fed control sham, *H*, pair-fed control OVX, *I*, EtOH-fed sham, and *J*, EtOH-fed OVX. *K*, Hepatic triglyceride levels, *L*, hepatic nonesterified fatty acid (NEFA) levels, *M*, hepatic cholesterol levels, and *N*, liver microsomal triglyceride transfer protein (*Mttp*) mRNA levels. Data are presented as mean ± SEM (n = 6–8). ∗*p* < 0.05, ∗∗*p* < 0.01, ∗∗∗*p* < 0.001, ∗∗∗*p* < 0.001, and ∗∗∗∗*p* < 0.0001 between indicated groups.

H&E and Oil Red-O staining revealed that lipid droplet accumulation was present in the livers of both EtOH-fed sham and OVX mice (Fig. 3, *A–D*, and *G–J*). Unexpectedly, the pathology score for steatosis from H&E staining was higher in the EtOH-fed OVX mice compared with the EtOH-fed sham mice but no evidence of necrosis or overt inflammation in any of the groups (Fig. 3, *E* and *F*). Interestingly, areas of low-grade steatohepatitis and small lipid droplet accumulation were observed in some of the EtOH-fed sham mice samples by the pathologist, which were absent in the EtOH-fed OVX group. Quantitative examination of the liver lipid levels revealed that EtOH ingestion significantly increased hepatic triglyceride levels to the same extent in both the sham and OVX mice (Fig. 3*K*). However, when compared with their respective controls, the EtOH-induced increase in hepatic triglycerides was greater in sham mice (∼2.8-fold) compared with the OVX mice (∼2.2-fold). Furthermore, hepatic NEFA levels were increased only in EtOH-fed sham mice (Fig. 3*L*). Conversely, cholesterol levels were significantly increased only in the livers of the EtOH-fed OVX mice, which was higher than the levels in the EtOH-fed sham mice (Fig. 3*M*). The basal mRNA levels of the microsomal triglyceride transfer protein (MTTP) involved in lipids transport out of the liver were significantly increased in the OVX control mice compared with the sham control mice (Fig. 3*N*). While EtOH increased the liver MTTP mRNA levels in the sham mice (1.6-fold), the levels significantly decreased by 50% in the OVX mice (Fig. 3*N*).

### Upregulation of major hepatic estrogen receptors may mediate alcohol-induced liver injury in mice

**Figure 4:**
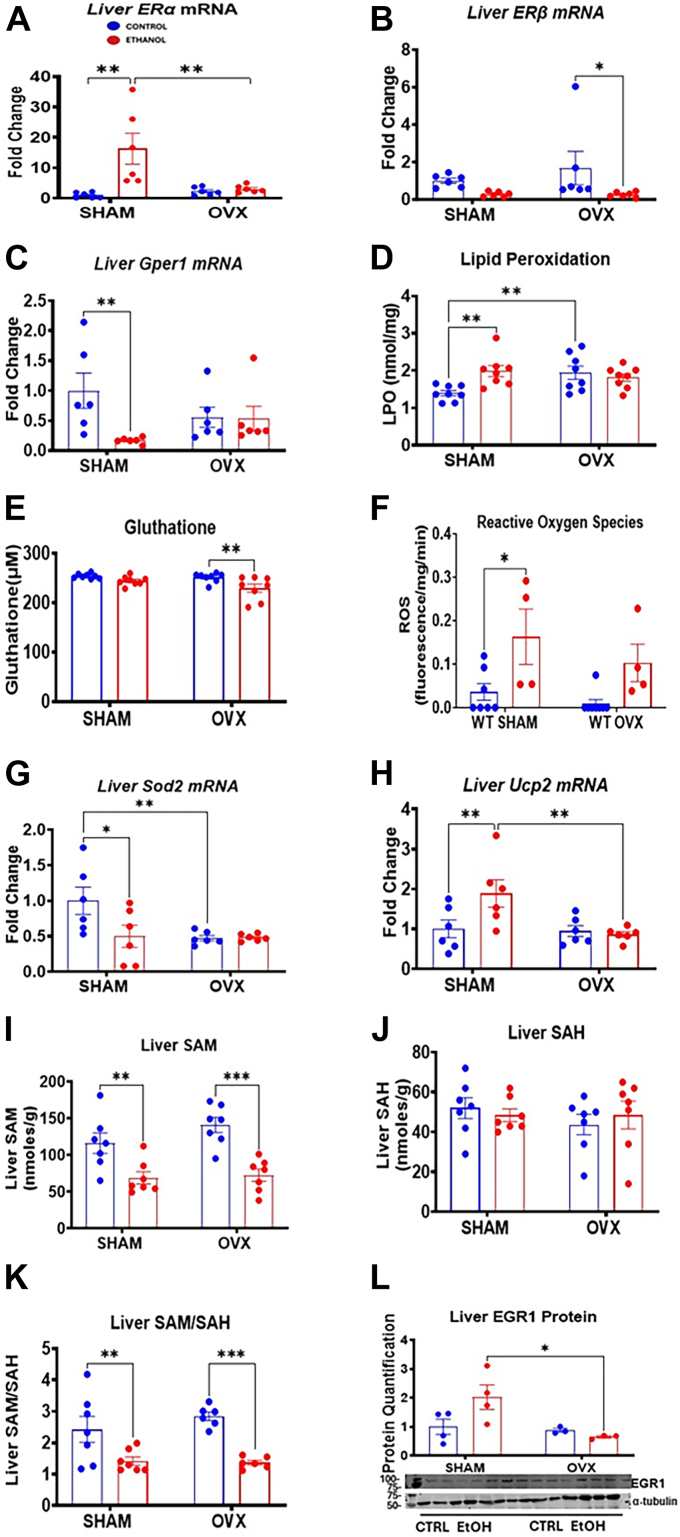
**Hepatic analysis of methylation index, lipid peroxidation, and hepatic genes involved in estrogen signaling, antioxidants, and Ucp2 gene expression.** Female C57BL/6N (WT) mice were subjected to either bilateral ovariectomy (OVX) or sham surgery. After recovery, the sham and OVX mice were pair-fed either a 5% ethanol (EtOH)-containing liquid diet or control diet for 10 days followed by a single oral dose of EtOH (5 g/kg, 30% EtOH solution), and mRNA and liver homogenate were prepared for the assays below described in the Experimental procedures section. *A*, Liver *Erα* mRNA expression, *B*, liver *Erb* mRNA, *C*, liver *Gper1* mRNA, and *D*, hepatic lipid peroxidation levels were quantified using the TBARS kit (ZeptoMetrix). *E*, Liver total GSH levels were quantified using a commercially available kit. *F*, Liver ROS, *G*, liver *Sod2* mRNA levels, *H*, Liver *Ucp2* mRNA levels, *I*, liver SAM levels, *J*, liver SAH levels, *K*, liver SAM/SAH ratio, and *L*, liver EGR1 protein. Data are presented as mean ± SEM (n = 3–6). ∗*p* < 0.05, ∗∗*p* < 0.01, and ∗∗∗*p* < 0.001 between indicated groups. EGR1, early growth response 1; ROS, reactive oxygen species; SAH, S-adenosylhomocysteine; SAM, S-adenosylmethionine; TBARS, thiobarbituric acid reactive substance.

Estrogen is associated with increased susceptibility to alcohol-induced liver injury in females (20). There are three main receptors that mediate estrogen signaling, namely, ERα, Erβ, and the G protein–coupled estrogen receptor 1 (Gper1) (27). Interestingly, 17 beta-estradiol (E2), the primary and most potent estrogen in humans, has affinity for all its three receptors with the strongest affinity for Erα and Erβ (27). We analyzed the effects of alcohol on these estrogen receptors important for estrogen signaling. Our results showed that alcohol significantly elevated ERα (*Era*) mRNA expression by ∼16.3-fold in the EtOH-fed sham mice compared with its control, whereas this increase was attenuated in EtOH-fed OVX mice (Fig. 4*A*). EtOH decreased ERβ (*Erb*) mRNA levels significantly only in EtOH-fed OVX mice (Fig. 4*B*). *Gper1* expression dropped by 83% in the EtOH-fed sham group but remained unchanged in OVX mice following EtOH treatment (Fig. 4*C*). Thiobarbituric acid reactive substance (TBARS) assay, which measures LPO as an indicator of oxidative stress, showed a significant increase (∼1.4-fold) in the EtOH-fed sham mice compared with controls, whereas control OVX mice displayed higher baseline LPO than sham control (Fig. 4*D*). Liver GSH levels on the other hand remained stable in the sham group, though a significant (∼8–10%) reduction occurred in EtOH-fed OVX mice (Fig. 4*E*). Consistent with increased LPO (Fig. 4*D*), hepatic reactive oxygen species (ROS) levels were significantly higher (4.5-fold) in the EtOH-fed sham mice but not in EtOH-fed OVX mice (Fig. 4*F*). Superoxide dismutase 2 (Sod2) is a mitochondrial enzyme involved in scavenging ROS and mitigating oxidative stress (28). Sod2 mRNA expression dropped by ∼40% to 50% only in the EtOH-fed sham group compared with its control, while remaining unchanged in OVX groups despite higher basal levels in sham controls (Fig. 4*G*). Unexpectedly, mitochondrial uncoupling protein 2 (*Ucp2*) mRNA expression was elevated by ∼1.9-fold in the EtOH-fed sham mice compared with its control and EtOH-fed OVX mice (Fig. 4*H*). Hepatic S-adenosylhomocysteine (SAH) levels remained unchanged in all groups; however, hepatic S-adenosylmethionine (SAM) and the SAM/SAH ratio were reduced in both EtOH-fed sham and OVX groups (Fig. 4, *I–K*). Moreover, there was also a 3.1-fold increase in early growth response 1 (EGR1) protein in the EtOH-fed sham mice compared with the EtOH-fed OVX mice (Fig. 4*L*).

### Alcohol ingestion in the presence of estrogen modulates the expression of alcohol-metabolizing genes

**Figure 5:**
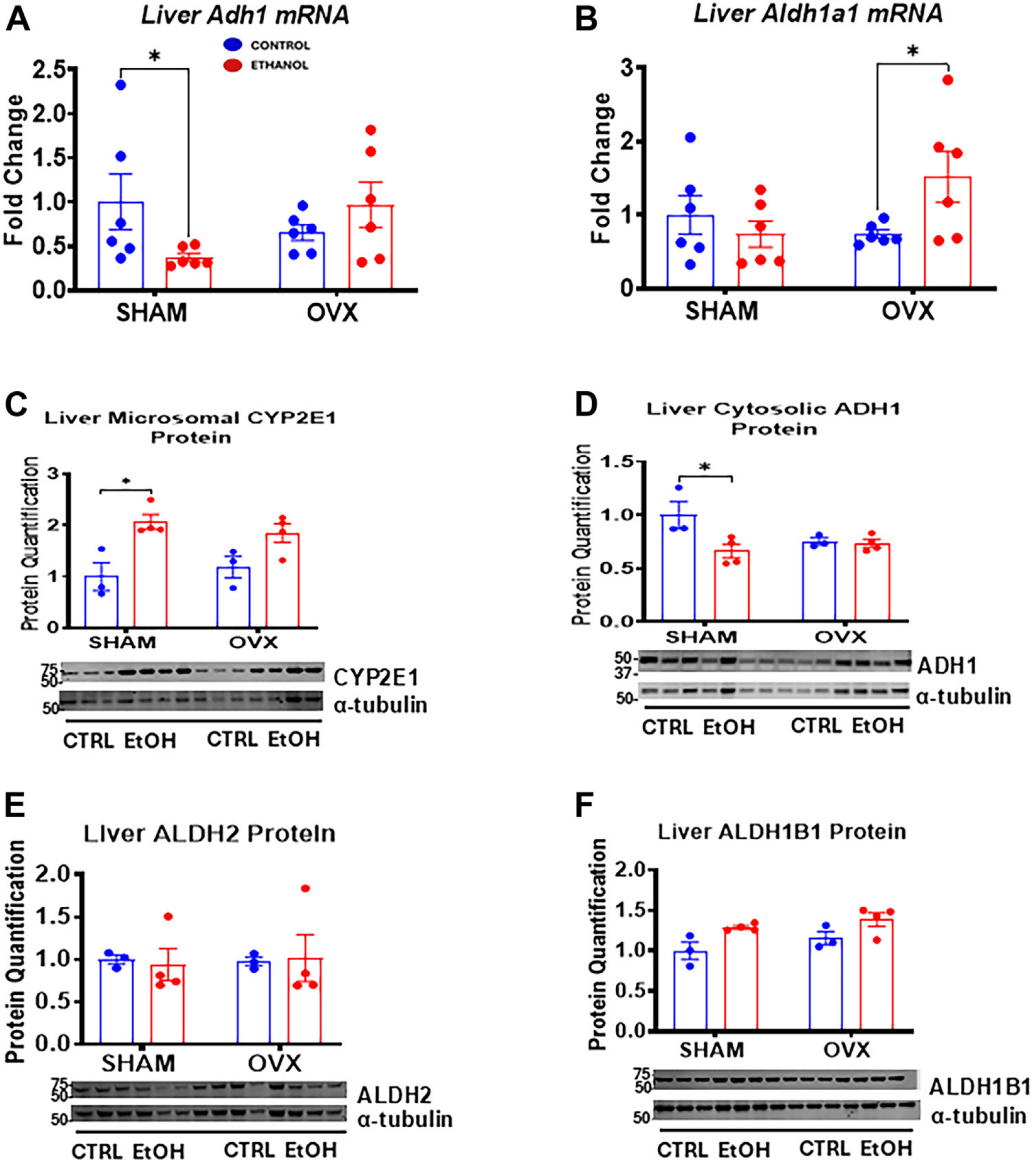
**Hepatic gene expression and immunoblot analysis of hepatic enzymes involved in ethanol (EtOH) metabolism.** Sham and OVX mice were pair-fed either a 5% EtOH-containing liquid diet or control diet for 10 days followed by a single oral dose of EtOH (5 g/kg, 30% EtOH solution), and mRNA, liver homogenate, cytosolic, and microsomal fractions were prepared for the assays described in the Experimental procedures section. *A*, Liver *Adh1* mRNA, *B*, liver *Aldh1a1* mRNA (n = 6), *C*, liver microsomal CYP2E1 protein, *D*, liver cytosolic ADH1 protein, *E*, liver ALDH2 protein, and *F*, liver ALDH1B1 protein (n = 3–4). Data are presented as mean ± SEM. ∗*p* < 0.05 between indicated groups. OVX, ovariectomy.

Since the liver metabolizes both alcohol and estrogen (29), we assessed how estrogen influences alcohol-metabolizing enzymes, as alcohol is typically discouraged in estrogen-related conditions like breast and endometrial cancers because of its effect on raising circulating estrogen levels (30, 31). *Adh1* mRNA levels significantly decreased by 63% in the EtOH-fed sham group, whereas they nonsignificantly increased by ∼1.5-fold in the EtOH-fed OVX group (Fig. 5*A*). Aldh1a1 mRNA, involved in cytosolic acetaldehyde metabolism, significantly increased ∼2.1-fold in EtOH-fed OVX mice (Fig. 5*B*). Protein data supported these findings, showing a significant decrease in ADH1 expression in EtOH-fed sham mice compared with its control, whereas aldehyde dehydrogenase 2 and ALDH1B1 (Aldehyde dehydrogenase 1 Family Member B1) protein levels were unchanged by EtOH (Fig. 5, *D–F*). We also examined CYP2E1, a marker for chronic alcohol exposure and oxidative stress, as it produces acetaldehyde and ROS. CYP2E1 protein levels significantly increased by 2-fold only in the EtOH-fed sham group compared with its control (Fig. 5*C*). The increased CYP2E1 levels in EtOH-fed sham mice (Fig. 5*C*) may promote oxidative stress indicative as increased ROS and LPO levels in EtOH-fed sham mice (Fig. 4, *D* and *F*). These findings suggest that estrogen combined with EtOH inhibits both ADH1 mRNA and protein expression and suppresses EtOH-induced upregulation of Aldh1a1 gene expression, affecting alcohol metabolism in the sham females.

### OVX reveals the role of estrogen in suppressing lipid oxidation after alcohol ingestion

**Figure 6:**
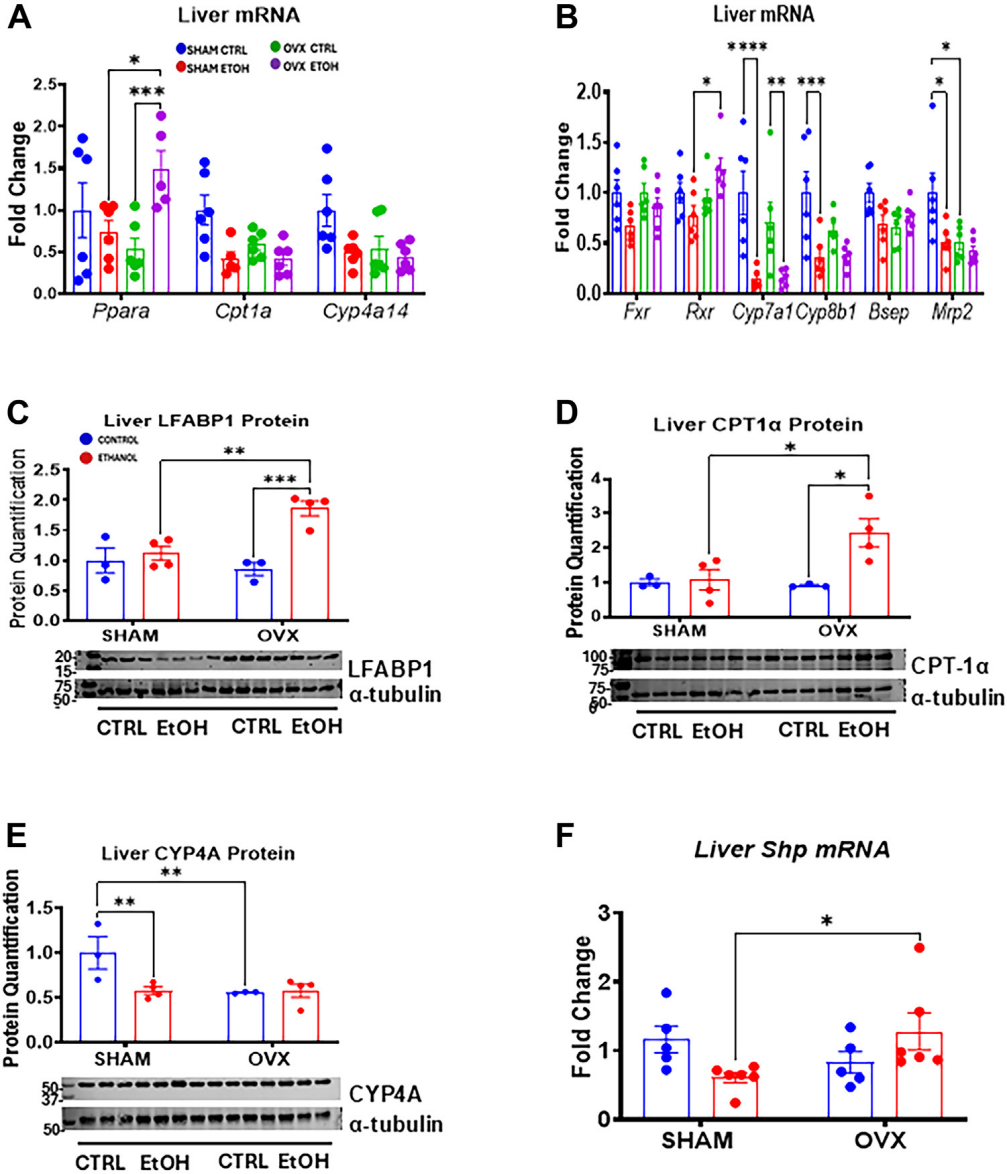
**Hepatic gene expression and immunoblot analysis of PPARα and its target genes involved in fatty acid β-oxidation and genes involved in bile acid synthesis.** Sham and OVX mice were pair-fed either a 5% ethanol (EtOH)-containing liquid diet or control diet for 10 days followed by a single oral dose of EtOH (5 g/kg, 30% EtOH solution) on day 11, and mRNA and liver homogenate were prepared for the assays below as described in the Experimental procedures section. *A*, Liver *Ppara*, *Cpt1a*, and *Cyp4a14* mRNA levels (n = 6). *B*, Liver *Fxr*, *Rxr*, *Cyp7a1*, *Cyp8b1*, *Shp*, *Bsep*, and *Mrp2* mRNAs (n = 6). *C*, Liver LFABP1 protein, *D*, liver CPT1A protein, and (*E*) liver CYP4A protein (n = 3–4), and (*F*) liver *Shp* mRNA (n = 5–6). The membrane initially probed for CPT1α (*D*) was subsequently stripped and reprobed with the CYP3A primary antibody (Fig. 8*C*), before finally being stripped and reprobed with α-tubulin antibody. Both the CPT1α and CYP3A bands were quantified and normalized using the same α-tubulin blot. Data are presented as mean ± SEM. ∗*p* < 0.05, ∗∗*p* < 0.01, ∗∗∗*p* < 0.001, and ∗∗∗*p* < 0.0001 between indicated groups. OVX, ovariectomy; PPARα, peroxisome proliferator–activated receptor alpha.

**Figure 8:**
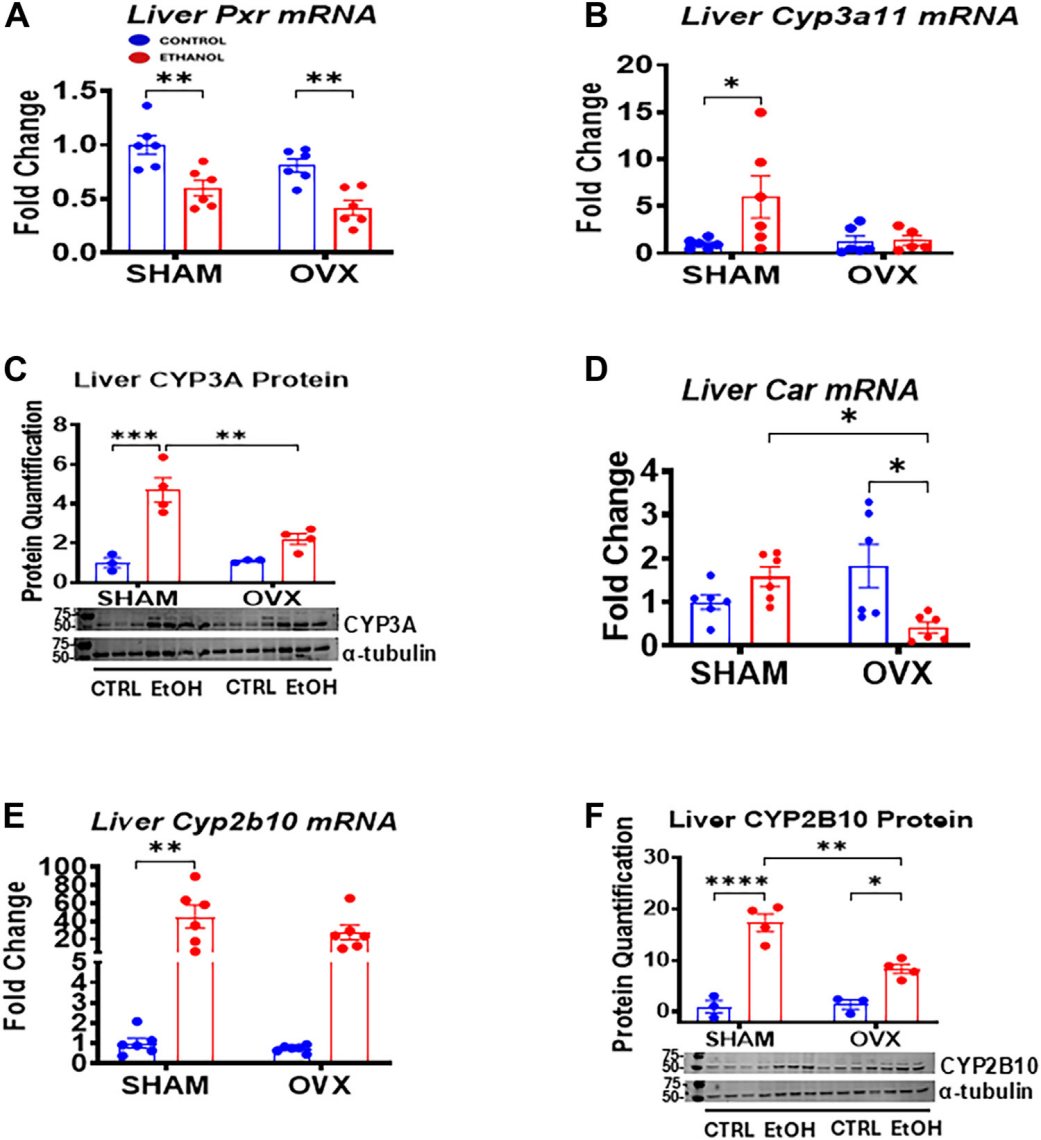
**Hepatic gene expression and immunoblot analysis of PXR, CAR, and their target gene mRNAs.** Sham and OVX mice were pair-fed either a 5% ethanol (EtOH)-containing liquid diet or control diet for 10 days followed by a single oral dose of EtOH (5 g/kg, 30% EtOH solution) on day 11, and mRNA and liver homogenate were prepared for the assays below as described in the Experimental procedures section. *A*, Liver *Pxr* mRNA, *B*, liver *Cyp3a11* mRNA, *C*, liver CYP3A11 protein, *D*, liver *Car* mRNA, *E*, liver *Cyp2b10* mRNA, and *F*, CYP2B10 protein levels. The membrane initially probed for CPT1α (Fig. 6*D*) was subsequently stripped and reprobed with the CYP3A primary antibody (*C*), before finally being stripped and reprobed with α-tubulin antibody. Both the CPT1α and CYP3A bands were quantified and normalized using the same α-tubulin blot. Data are presented as mean ± SEM (n = 3–6). ∗*p* < 0.05, ∗∗*p* < 0.01, ∗∗∗*p* < 0.001, and ∗∗∗∗*p* < 0.0001 between indicated groups. CAR, constitutive androstane receptor; OVX, ovariectomy; PXR, pregnane X receptor.

Peroxisome proliferator–activated receptor alpha (PPARα) and its target genes, *Cyp4a14* and *Cpt1a*, are crucial for mitochondrial β-oxidation, enabling fatty acids to convert into acetyl-CoA for energy (32). Our findings show that estrogen absence notably upregulates Pparα and its target genes, key regulators of mitochondrial fatty acid β-oxidation (Fig. 6). Specifically, EtOH-fed OVX mice exhibited a ∼2.8-fold increase in *Ppara* mRNA levels compared with both OVX control and EtOH-fed sham mice (Fig. 6*A*). While *Cpt1* and *Cyp4a14* mRNA levels decreased by 50% to 60% in EtOH-fed sham mice, this difference was not significant, and the levels remained stable across the OVX groups (Fig. 6*A*). Western blot results also showed a significant increase in the Pparα target, FABP1 (fatty acid binding protein 1) protein expression in the EtOH-fed OVX mice compared with its control and EtOH-fed sham mice (Fig. 6*C*). The basal CYP4A protein levels were significantly lower in the OVX mice compared with the sham controls (Fig. 6*E*). EtOH significantly decreased the CYP4A protein by ∼43% in the EtOH-fed sham mice but remained unchanged in OVX mice, consistent with *Cyp4a14* mRNA expression (Fig. 6, *A* and *E*). Notably, CPT1α protein expression increased ∼2.3-fold in EtOH-fed OVX mice compared with EtOH-fed sham mice (Fig. 6*D*). The membrane used to probe for CPT1α protein was subsequently stripped and reprobed with the primary antibody against CYP3A (Fig. 8*C*) and finally stripped and reprobed with α-tubulin antibody. Bands from the CPT1α (Fig. 6*D*) and CYP3A (Fig. 8*C*) protein were quantified and normalized to the same α-tubulin blot. Liver mRNA analysis of bile acid metabolism and transport genes revealed a ∼33% nonsignificant reduction in *Fxr* gene expression in EtOH-fed sham mice but not in the EtOH-fed OVX mice (Fig. 6*B*). In addition, *Rxr* mRNA levels were significantly higher in EtOH-fed OVX mice compared with EtOH-fed sham mice (Fig. 6*B*). Although *Cyp7a1* and *Cyp8b1* mRNA expressions decreased by ∼86% and ∼64%, respectively, in EtOH-fed shams, this pattern in EtOH-fed OVX groups was only statistically significant for *Cyp7a1* mRNA (Fig. 6*B*). Bile acid transporter gene, *Shp* mRNA levels were reduced by ∼40% in EtOH-fed sham mice compared with OVX counterparts (Fig. 6*F*), whereas *Mrp2* mRNA significantly decreased by ∼50% in EtOH-fed sham group, remaining unchanged in EtOH-fed OVX mice (Fig. 6*B*). Surprisingly, the basal *Mrp2* mRNA was significantly lower in the OVX controls compared with the sham controls (Fig. 6*B*). These data suggest that estrogen combined with EtOH downregulates lipid oxidation genes and impacts on lipolysis *via* β-oxidation.

### Chronic binge alcohol intake promotes fat accumulation in the liver of sham mice through the induction of lipogenic genes and proteins in the presence of estrogen

**Figure 7:**
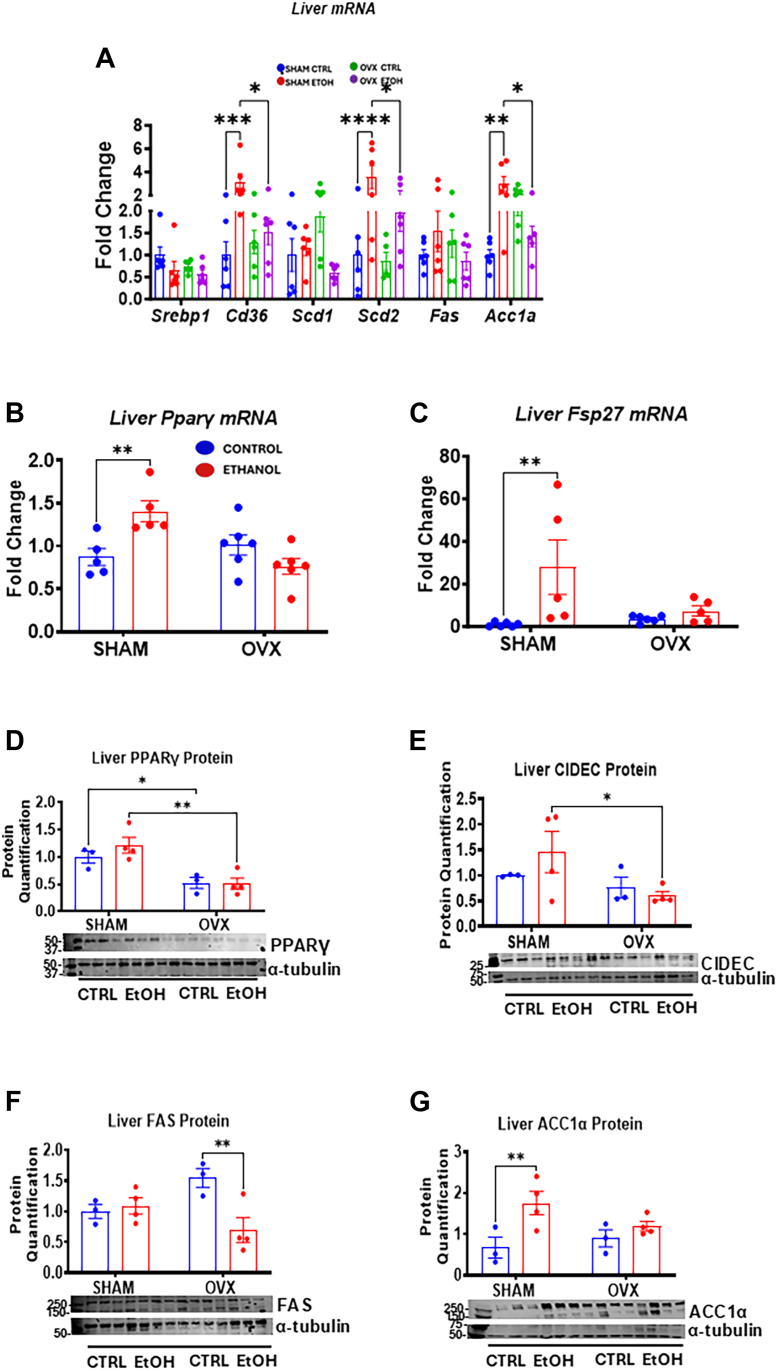
**Hepatic gene expression and immunoblot analysis of lipogenic enzymes.** Sham and OVX mice were pair-fed either a 5% ethanol (EtOH)-containing liquid diet or control diet for 10 days followed by a single oral dose of EtOH (5 g/kg, 30% EtOH solution) on day 11, and mRNA and liver homogenate were prepared for the assays as described in the Experimental procedures section. *A*, Liver *Srebp1*, *Cd36*, *Scd1*, *Scd2*, *Fas*, and *Acc1a* mRNAs (n = 5–6). *B*, Liver *Pparγ* mRNA and (*C*) liver *Fsp27*/*Cidec* mRNA (n = 6). *D*, Liver PPARγ protein, *E*, liver FSP27/CIDEC protein, *F*, liver FAS protein, and (*G*) liver ACC1α protein (n = 3–4). Data are presented as mean ± SEM. ∗*p* < 0.05, ∗∗*p* < 0.01, ∗∗∗*p* < 0.001, and ∗∗∗∗*p* < 0.0001 between indicated groups. OVX, ovariectomy; PPARγ, peroxisome proliferator–activated receptor gamma.

Sterol regulatory element-binding protein-1c (SREBP-1c) regulates lipogenic genes essential for lipid metabolism, such as acyl-CoA carboxylase (Acc1a), fatty acid synthase (Fas), stearoyl-CoA desaturase 1 (Scd1), and Scd2 (33). While hepatic *Srebp-1c* mRNA expression remained unaffected in the EtOH-treated groups (Fig. 7*A*), estrogen recycling in sham mice significantly increased *Acc1a* mRNA (3.0-fold) and ACC1α protein (2.6-fold) only in the EtOH-fed sham group compared with its pair-fed control (Fig. 7*G*). However, in EtOH-fed OVX mice, EtOH ingestion reduced *Scd1* mRNA by 70% with no effect on *Fas* mRNA levels but significantly decreased the FAS protein (∼60%), compared with its control (Fig. 7, *A* and *F*). The SREBP-1c target gene *Scd2* mRNA was significantly elevated by 3.6-fold in EtOH-fed sham mice compared with both its pair-fed control and EtOH-fed OVX mice (Fig. 7*A*). We further examined the lipogenic gene PPARγ and its targets, including fat-specific protein 27 (*Fsp27*)*/Cidec* and *Cd36*, which contribute to triglyceride accumulation in steatotic livers. The PPARγ protein levels were significantly reduced by 50% in OVX mice compared with sham groups (Fig. 7*D*). Meanwhile, the mRNA levels of *Pparγ*, along with *Fsp27* mRNA, FSP27/CIDEC protein, and *Cd36* mRNA, were all significantly increased in EtOH-fed sham mice but not in the EtOH-fed OVX mice (Fig. 7, *A*–*C*, and *E*), indicating increased lipogenesis in the EtOH-fed sham group.

### PXR–constitutive androstane receptor nuclear receptor and their downstream targets are directly implicated in this pathology

It has been well established that either activation or inhibition of PXR plays a significant role in the development of steatosis in EtOH-fed mice (34). Our results in Figure 8*A* indicated that EtOH significantly inhibited *Pxr* mRNA levels in the livers of both sham and OVX groups. Surprisingly, the PXR target gene, *Cyp3a11* mRNA, and CYP3A protein levels were increased by ∼5.9-fold and ∼4.7-fold, respectively, in the EtOH-fed sham mice compared with their respective controls with no significant increases in the EtOH-fed OVX mice (Fig. 8, *B* and *C*). Even though *Car* mRNA levels were not significantly affected by EtOH treatment in the sham mice, the levels were significantly decreased by EtOH treatment in the OVX mice with a significant decrease in *Car* gene expression between the EtOH-fed sham and EtOH-fed OVX mice (Fig. 8*D*). Interestingly, the constitutive androstane receptor (CAR) target gene, *Cyp2b10* mRNA, implicated in EtOH-induced hepatotoxicity (35) was significantly increased by EtOH ingestion in both the sham (∼45-fold) and OVX groups (∼37-fold) leading to a more significant increase in the CYP2B10 protein levels in the EtOH-fed sham mice than in the EtOH-fed OVX mice (Fig. 8, *E* and *F*).

### Nrf2–Hmox1 and AhR–Nqo1 signaling pathways protect ovariectomized mice against EtOH-induced liver injury

**Figure 9:**
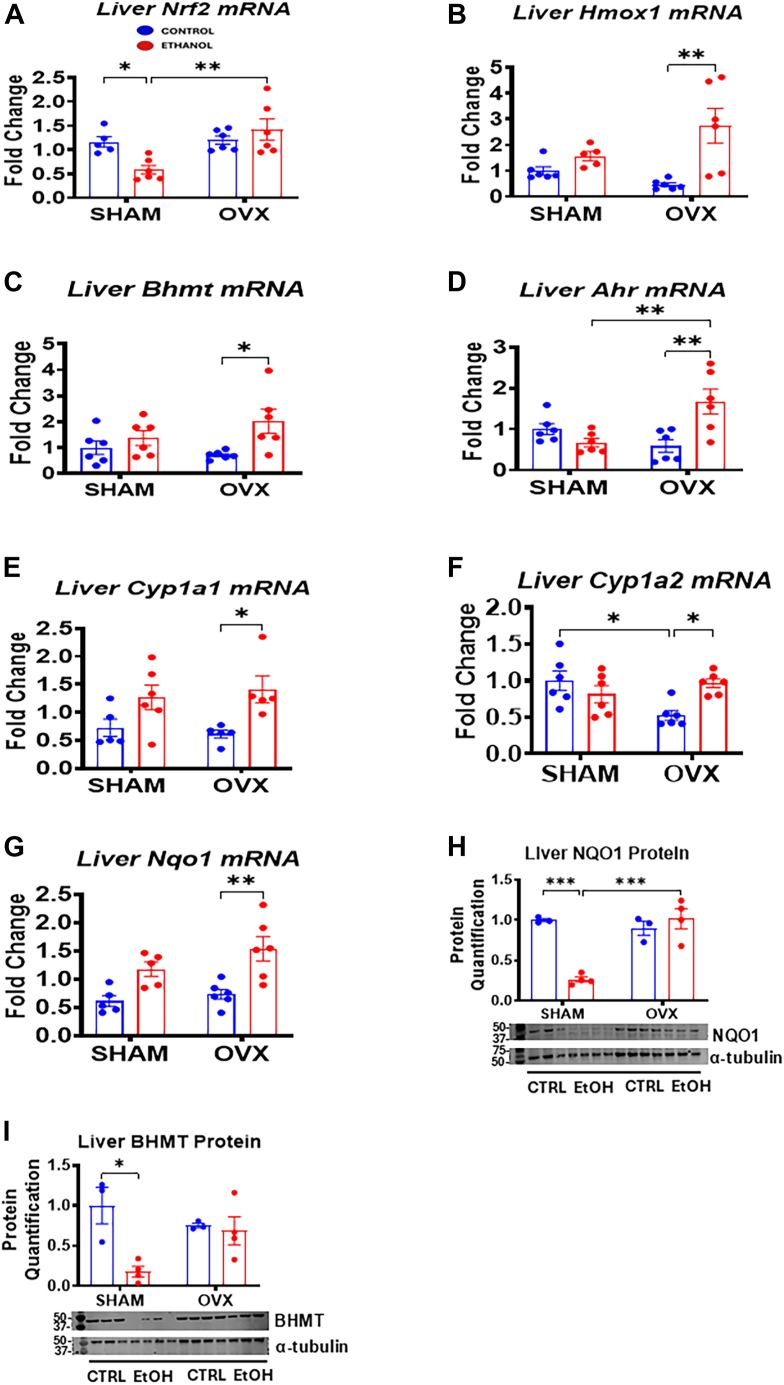
**Hepatic gene expression and immunoblot analysis of Nrf2–Hmox1 and Ahr–NQO1 signaling pathway in sham and ovariectomized (OVX) mice.** Sham and OVX mice were pair-fed either a 5% ethanol (EtOH)-containing liquid diet or control diet for 10 days followed by a single oral dose of EtOH (5 g/kg, 30% EtOH solution) on day 11, and mRNA and liver homogenate were prepared for the assays as described in the Experimental procedures section. *A*, Liver *Nrf2* mRNA, *B*, liver *Hmox1* mRNA, *C*, liver *Bhmt* mRNA, *D*, liver *AhR* mRNA, *E*, liver *Cyp1a1* mRNA, *F*, liver *Cyp1a2* mRNA, *G*, liver *Nqo1* mRNA, *H*, liver NQO1 protein, and *I*, liver BHMT protein. Data are presented as mean ± SEM (n = 3–6). ∗*p* < 0.05, ∗∗*p* < 0.01, and ∗∗∗*p* < 0.001, between indicated groups. BHMT, betaine-homocysteine *S*-methyltransferase.

NRF2 and AhR are key transcription factors involved in liver protection (36, 37). AhR is a ligand-activated transcription factor abundant in hepatocytes, whereas NRF2 is activated by xenobiotic and endogenous stress, promoting cytoprotective gene expression (38). Both factors have been shown to mitigate liver injury from acute EtOH exposure through the activation of protective target genes (36, 39, 40, 41). In our study, we observed a significant downregulation of hepatic *Nrf2* gene (about 50%) in EtOH-fed sham mice, whereas the levels in EtOH-fed OVX mice remained unchanged and significantly higher than in the EtOH-fed sham mice (Fig. 9*A*). Correspondingly, the mRNA levels of NRF2 target genes, *Hmox1* and *Bhmt*—both antioxidants—increased significantly in the OVX mice following EtOH treatment but not in sham mice (Fig. 9, *B* and *C*). In addition, betaine-homocysteine S-methyltransferase (BHMT) protein levels decreased significantly in EtOH-fed sham mice but remained unchanged in EtOH-fed OVX mice (Fig. 9*I*). We also found a significant increase in liver *Ahr* mRNA levels (2.9-fold) in EtOH-fed OVX mice compared with both its pair-fed control and EtOH-fed sham mice (Fig. 9*D*). The AhR target genes, *Cyp1a1* and *Cyp1a2*, were significantly upregulated by 2.3-fold and 1.8-fold, respectively, in the EtOH-fed OVX mice compared with its pair-fed control but not in the EtOH-fed sham mice (Fig. 9, *E* and *F*). To investigate the involvement of NQO1 signaling in AhR upregulation, we measured *Nqo1* mRNA and NQO1 protein levels and found that the *Nqo1* mRNA levels were significantly elevated in EtOH-fed OVX mice by ∼2.1-fold, whereas the NQO1 protein remained unchanged (Fig. 9, *G* and *H*). Conversely, the NQO1 protein expression was reduced by 74% in the EtOH-fed sham group compared with its pair-fed control (Fig. 9*H*). Furthermore, after EtOH ingestion, the NQO1 protein levels were significantly lower in the EtOH-fed sham mice compared with the EtOH-fed OVX mice (Fig. 9*H*). These findings suggest that the combined NRF2–HMOX1 (heme oxygenase 1) and AhR–NQO1 signaling pathways may contribute to the hepatoprotection observed in EtOH-fed OVX mice against liver injury by activating antioxidant genes and proteins that suppress oxidative stress.

### Chronic binge alcohol feeding induces more significant inflammation in the liver and adipose tissue of female sham mice than the ovariectomized mice

**Figure 10:**
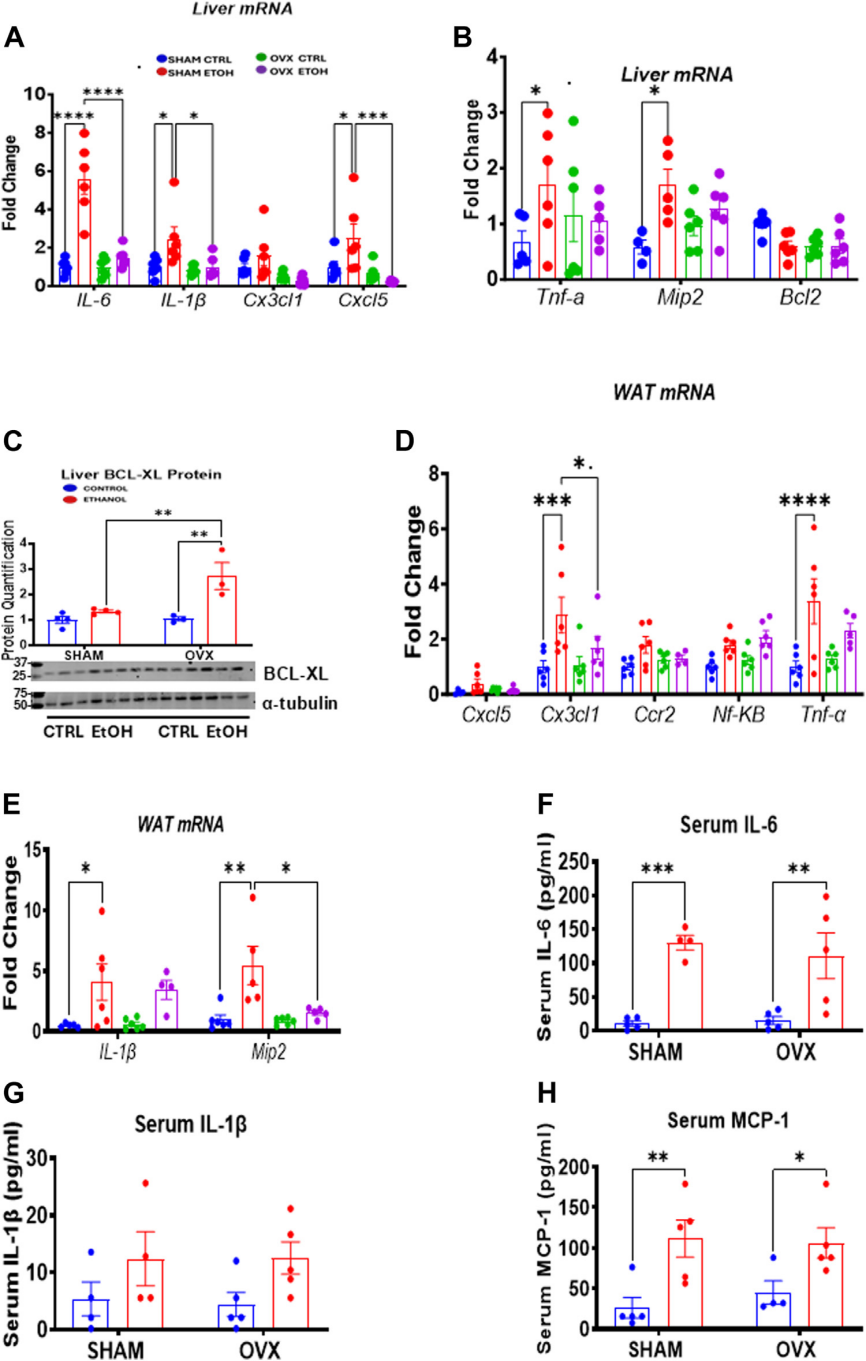
**Hepatic and adipose tissue inflammatory and chemokine gene expression.** Sham and OVX mice were pair-fed either a 5% ethanol (EtOH)-containing liquid diet or control diet for 10 days followed by a single oral dose of EtOH (5 g/kg, 30% EtOH solution) on day 11, and mRNA from liver and pgWAT as well as liver homogenate and serum were prepared for the assays below as described in the Experimental procedures section. *A*, Liver *Il6*, *Il1B*, *Cx3cl1*, and *Cxcl5* mRNAs. *B*, Liver *Tnfa*, *Mip2*, and *Bcl2* mRNAs, *C*, liver BCL-XL protein, *D*, pgWAT *Cxcl5*, *Cx3cl1*, *Ccr2*, *Nfkβ*, and *Tnfa* mRNAs, *E*, WAT *Il1β* and *Mip2* mRNAs. *F*, Serum IL6 levels, *G*, serum IL-1β levels, and (*H*) serum MCP1 levels. Data are presented as mean ± SEM (n = 3–6). ∗*p* < 0.05, ∗∗*p* < 0.01, ∗∗∗*p* < 0.001, and ∗∗∗∗*p* < 0.0001 between indicated groups. BCL-XL, B-cell lymphoma-extra large; OVX, ovariectomy; pgWAT, perigonadal white adipose tissue.

Oxidative stress and inflammation play critical roles in the pathology of ALD (42). Inhibition of AhR signaling has been linked to disrupted redox balance in the liver, resulting in increased oxidative stress, apoptosis, steatosis, and inflammation (43). In our study, we assessed the mRNA expression of proinflammatory cytokines, chemokines, and apoptotic genes in the liver and/or pgWAT. We observed significant increases in hepatic mRNA levels of proinflammatory cytokines in EtOH-fed sham mice compared with pair-fed controls: *Il6* (5.6-fold), *Il1b* (2.5-fold), Tnfα (2.6-fold), *Mip2* (Macrophage Inflammatory Protein 2; 2.9-fold), and *Cxcl5* (2.5-fold) (Fig. 10, *A* and *B*). In addition, hepatic *Il6* (3.7-fold), *Il1b* (2.53-fold), and *Cxcl5* (10.7-fold) mRNA levels were significantly higher in the EtOH-fed sham group than in the EtOH-fed OVX group (Fig. 10*A*). EtOH ingestion had no significant effect on the mRNA levels of the chemokine *Cx3cl1* (chemokine [C-X3-C motif] ligand 1) and the antiapoptotic *Bcl2* in the livers of both sham and OVX mice (Fig. 10, *A* and *B*). In contrast, the protein expression of another antiapoptotic protein, B-cell lymphoma-extra large (BCL-XL), was significantly increased (2.7-fold) in the livers of the EtOH-fed OVX mice, and the levels were about 2.4-fold higher compared with the EtOH-fed sham mice (Fig. 10*C*). Also, in the pgWAT, we detected significant increases in *Cx3cl1* (2.9-fold), *Tnfα* (3.4-fold), *Il1b* (8.1-fold), and *Mip2* (5.4-fold) mRNA levels in EtOH-fed sham mice (Fig. 10, *D* and *E*). Moreover, serum multiplex assay revealed statistically significant increases in IL-6 and MCP-1 levels for both EtOH-fed groups; however, the increases were higher in the EtOH-fed sham group (4.2- to 13.0-fold) than in the EtOH-fed OVX group (2.4- to 7.4-fold) (Fig. 10, *F* and *H*). We also found that the serum IL-1β levels in both EtOH-fed groups tended to increase compared with their respective controls (Fig. 10*G*). These findings suggest that chronic binge EtOH consumption in the presence of estrogen enhances inflammatory markers in serum, liver, and adipose tissue to a greater extent than in the absence of estrogen.

### Expression of Er**α**, Fgf21, and Pxr and Car target genes as well as lipogenic genes in the pgWAT after chronic binge EtOH feeding

**Figure 11:**
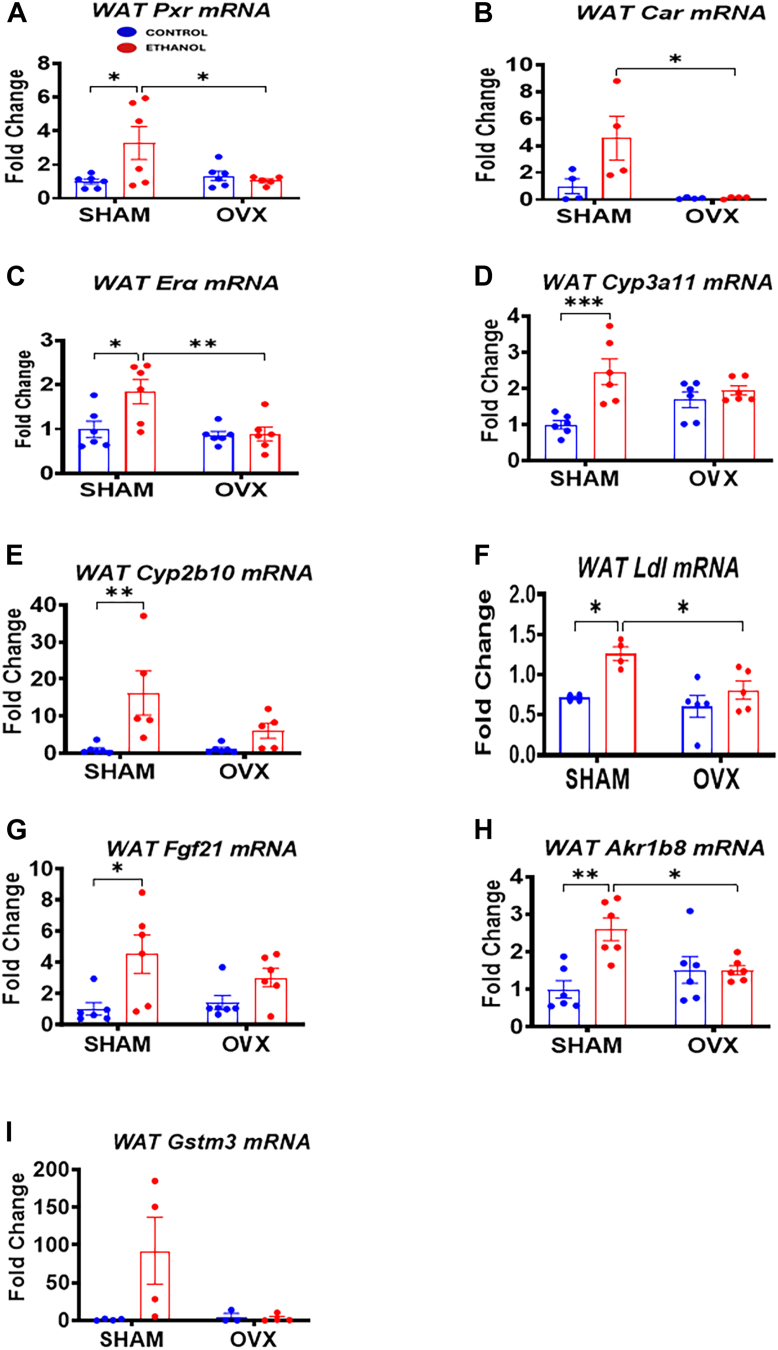
**Perigonadal white adipose tissue (pgWAT or WAT) *Pxr* and *Car* and their target genes as well as lipogenic and antioxidant genes.** Sham and OVX mice were pair-fed either a 5% ethanol (EtOH)-containing liquid diet or control diet for 10 days followed by a single oral dose of EtOH (5 g/kg, 30% EtOH solution) on day 11, and pgWAT mRNA were prepared for the assays below as described in the *Experimental procedures* section. *A*, WAT *Pxr* mRNA expression, *B*, WAT *Car* mRNA, *C*, WAT *Era* mRNA, *D*, WAT *Cyp3a11* mRNA, *E*, WAT *Cyp2b10* mRNA, *F*, WAT Ldl mRNA, *G*, WAT Fgf21 mRNA, *H*, WAT Akr1b8 mRNA, and *I*, WAT Gstm3 mRNA. Data are represented as mean ± SEM (n = 4–6). ∗*p* < 0.05, ∗∗*p* < 0.01, and ∗∗∗*p* < 0.001 between indicated groups. OVX, ovariectomy.

Adipose tissue plays a crucial role in alcohol-induced fatty liver disease (44) with PXR expression in white adipose tissue having been previously reported (45). Recent studies indicate that chronic alcohol consumption can trigger inflammation in adipose tissue, potentially leading to liver damage (46). Having confirmed increased inflammation in the adipose tissue of EtOH-fed sham mice (Fig. 10), we examined the expression of genes related to hepatotoxicity and hepatoprotection. We found significant increases in the mRNA levels of *Pxr* (3.3-fold) and its target gene *Cyp3a11* (2.5-fold) in EtOH-fed sham mice compared with their respective controls (Fig. 11, *A* and *D*). Similarly, increases in *Car* mRNA (4.6-fold) and its target gene *Cyp2b10* mRNA (16.2-fold) were also found in the EtOH-fed sham mice but not in the EtOH-fed OVX group (Fig. 11, *B* and *E*). Induction of *Fgf21* in adipose tissue contributes to alcohol-induced lipolysis and exacerbates hepatic steatosis (47). Indeed, pgWAT *Fgf21* mRNA levels significantly increased 4.5-fold in the EtOH-fed sham mice but not in EtOH-fed OVX mice (Fig. 11*G*). In addition, we observed a 1.8-fold increase in the mRNA expression of the major estrogen receptor, *Era*, in the EtOH-fed sham group compared with its pair-fed control and EtOH-fed OVX groups (Fig. 11*C*). Notably, lipogenic genes *Akr1b8* (Aldo-keto Reductase Family 1, member B8) and *Ldl*, which regulate cholesterol levels and are associated with metabolic dysfunction in adipose tissue, were significantly upregulated in the EtOH-fed sham mice (Fig. 11, *F* and *H*). Unexpectedly, Gstm3 mRNA levels were significantly elevated in the EtOH-fed sham group compared with its pair-fed control (Fig. 11*I*), potentially reflecting a compensatory mechanism to mitigate liver oxidative stress from EtOH ingestion.

## Discussion

**Figure 12:**
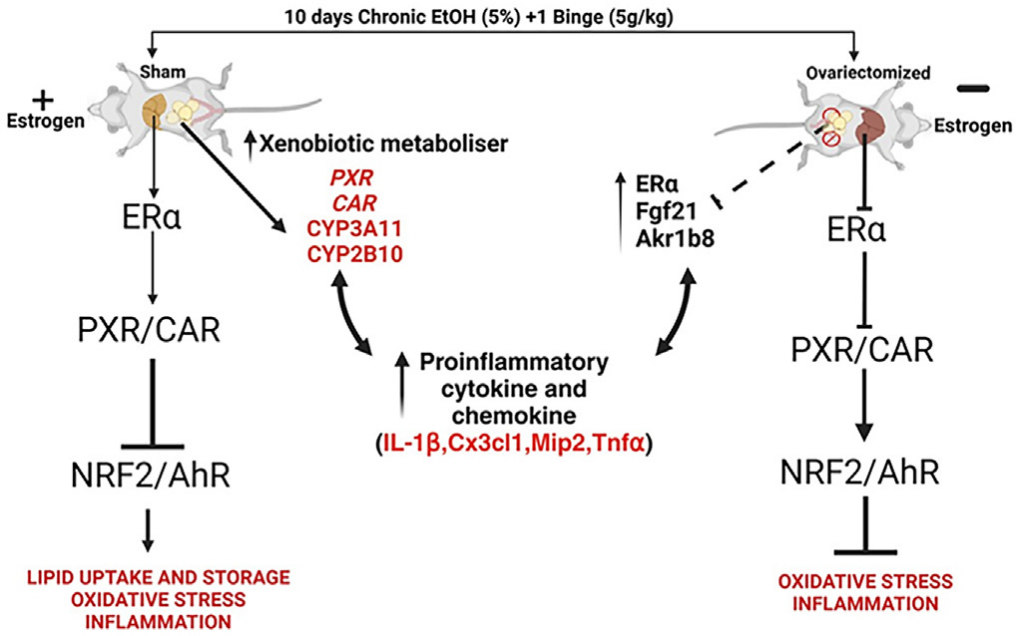
**Scheme highlighting the main findings of the study.** Activation (or inhibition) of estrogen receptor α (ERα) by estrogen causes phenotypic changes in the liver and perigonadal white adipose tissue (pgWAT) of mice fed ethanol, and these lead to downstream secondary and tertiary effects involving altered pregnane X receptor (PXR), nuclear factor erythroid 2-related factor (NRF2), and aryl hydrocarbon receptor (AhR) signaling that are likely because of an increase in ROS that activates or inhibits NRF2, and the production of endogenous ligands for PXR and AhR that activate these receptors. Together, these results suggest the existence of an ERα–PXR–NRF2 signaling axis in liver and pgWAT, which contributes to sex differences seen in ALD and demonstrates a previously unrecognized ERα signaling pathway, which contributes to sex differences in ALD. ALD, alcohol-associated liver disease; ROS, reactive oxygen species.

Women experience greater susceptibility to alcohol-induced liver injury than men at all levels of alcohol consumption (48), with estrogen implicated as a critical factor (18). However, the mechanisms by which estrogen exacerbates ALD in females remain unclear. Our study demonstrates that OVX in female mice mitigates EtOH-induced hepatotoxicity by suppressing ERα, PXR, and CAR target genes linked to EtOH-induced liver injury, whereas inducing the antioxidant transcription factor *Nrf2*, its target genes *Hmox1 and Bhmt* and *AhR*-NQO1 signaling, as well as the antiapoptotic BCL-XL protein, which together protected OVX mice against alcohol-related liver injury. These changes collectively reduced oxidative stress, inflammation, and apoptosis but increased EtOH metabolism, offering mechanistic insights into estrogen’s role in ALD as illustrated in Fig. 12.

Consistent with prior studies linking estrogen deficiency to obesity (49, 50), OVX mice demonstrated increased body and adipose tissue weight independent of food intake, validating our surgical procedure. To assess the role of satiety hormones, we examined two adipokines, leptin and adiponectin, both of which can modulate hepatic lipid homeostasis leading to reduction in liver lipid accumulation (51). Leptin levels are normally proportional to fat mass (52). Interestingly, chronic alcohol exposure decreases adipose tissue mass leading to decreased leptin levels but increased hepatic lipid accumulation (51, 53). Therefore, the elevated leptin mRNA levels we observed in pgWAT, especially in our EtOH-fed OVX mice, may reflect compensatory mechanisms to suppress hepatic steatosis. Adiponectin plays a complex role in liver disease, with elevated levels often associated with severe liver damage and fibrosis because of its metabolic and inflammatory interactions (44, 54, 55). In this study, pgWAT adiponectin mRNA levels were increased in EtOH-fed sham mice alongside higher BAC, LPO, oxidative stress, and ALT levels, indicating more severe liver damage despite comparable adiposity. In agreement with increased liver injury seen in the EtOH-fed sham mice, we also observed significant increases in the mRNA and/or protein levels of the major estrogen receptor ERα, PXR, and CAR target genes, but inhibition of *Nrf2* and its target genes encoding NQO1 and BHMT in liver and/or adipose tissue, further implicating estrogen in the amplification of oxidative stress and inflammation in ALD.

Alcohol-induced liver damage primarily arises from oxidative stress and inflammation (3). Recent research highlights the role of adipose tissue in this process, with chronic alcohol intake inducing inflammation, adipose tissue lipolysis, and increase in FGF21 expression contributing to liver dysfunction through an increase in NEFA circulation (44, 46). Adipose tissue, particularly, pgWAT, has been found to play a crucial role in ALD development because of its interaction with liver and the release of fatty acids from lipolysis leading to lipid accumulation. In energy-deficient states, pgWAT controls lipid storage and energy balance. This equilibrium is usually interrupted during chronic alcohol exposure, uncoupling lipolysis from thermogenesis, and increasing the amounts of fatty acids that accumulate in the liver, thus worsening steatosis. Reports indicate that chronic binge EtOH administration induces FGF21 in pgWAT, which increases lipolysis and lipid accumulation in the liver (56). In addition, changes in pgWAT-secreted adipokines such as leptin and adiponectin during chronic alcohol consumption can increase inflammation and contribute to the development of ALD (57). These observations prompted us to examine estrogen's impact on nuclear receptors and their targets in pgWAT in our EtOH-fed sham and OVX mice. Mirroring our liver results, estrogen influenced metabolic, detoxification, and inflammatory gene expression in pgWAT, exacerbating alcohol-induced liver damage.

A surprising observation in this study was that both the liver FGF21 protein and the pgWAT *Fgf21* mRNA were significantly elevated only in the EtOH-fed sham mice, consistent with the liver injury we observed. FGF21 is a mediator of liver metabolism and a marker of mitochondrial dysfunction and cirrhosis (26), a potential biomarker for predicting ALD severity. Activation of FGF21 may have contributed to the inhibition of *Cyp7a1* independent of the FXR–SHP pathway (58). In addition, Cyp7a1 deficiency (59), linked to increased EtOH-induced inflammation, aligns with elevated LPO, and increased inflammatory markers including *IL-1β*, *Tnfα*, and *CCL2* in liver and adipose tissue of the EtOH-fed sham mice. These results suggest that estrogen deficiency in OVX mice suppresses EtOH-induced FGF21 upregulation and possibly protects against EtOH-induced inflammation, ROS generation, oxidation stress, and hepatotoxicity (47). In agreement with increased FGF21 expression, we showed an increase in hepatic NEFA levels in the EtOH-fed sham mice (47).

Previous reports indicate that estrogen treatment alleviates steatosis, whereas treatment with tamoxifen, an estrogen receptor antagonist, promotes fatty liver (20, 22, 60). Moreover, in a previous report, we observed more pronounced lipid accumulation in male C57BL/6J mice than female C57BL/6J mice after chronic EtOH administration suggesting that estrogen protects against induction of hepatic steatosis (61). In the current study, while EtOH induced lipid accumulation in both sham and OVX mice, the pathology score for steatosis was higher in the EtOH-fed OVX mice. However, hepatic NEFA levels were significantly increased by EtOH-fed sham mice. Therefore, it was of interest to investigate the effect of EtOH and estrogen on genes involved in lipid homeostasis. Unexpectedly, CYP4A, a PPARα target controlling fatty acid β-oxidation, was reduced in the livers of the EtOH-fed sham mice, suggesting estrogen signaling inhibits lipid oxidation. In addition, hepatic lipogenic genes, including SREBP-1c, target *Scd1* and acetyl-CoA carboxylase 1α (*Acc1α*), and PPARγ and its targets *Cd36* and *Fsp27/Cidec*, as well as EGR1 protein, were all elevated in our EtOH-fed sham mice, further implying that estrogen signaling promotes EtOH-induced lipogenesis while blocking fatty acid transport into mitochondria for beta oxidation. ACC1α produces malonyl-CoA, which inhibits CPT1α and serves as a precursor for fatty acid synthesis (62). OVX mice, devoid of estrogen, had elevated *Pparα* mRNA, and protein levels of Pparα targets CPT1 and LFABP1, which supports lipid oxidation (63). However, MTTP, critical for lipid export, was reduced in EtOH-fed OVX mice, which can lead to increased lipid accumulation and possibly account for the higher pathology scores for steatosis in these mice (64). Overall, it is interesting to note that lipid accumulation was comparable between the sham and OVX mice, after EtOH ingestion, suggesting that lipid accumulation was not a major determinant of hepatoxicity seen in the EtOH-fed sham mice as previously observed after EtOH exposure in the liver-specific regulatory-associated protein of mTOR knockout mice (65). Considered together, our results indicate different mechanisms for EtOH-induced steatosis in sham and OVX mice.

Estrogen and alcohol are metabolized in the liver, with alcohol known to elevate circulating estrogen and ERα levels (29). Given that EtOH-fed sham mice had markedly elevated BAC, we explored the impact of alcohol-metabolizing enzymes. EtOH is metabolized by ADH into its toxic metabolite, acetaldehyde, which ALDHs further metabolize into acetate (16, 66). Acetate enters the tricarboxylic acid cycle as acetyl-CoA, a precursor for lipogenesis. While we did not measure the NAD+/NADH ratio in this study, evidence indicates that alcohol metabolism disrupts the NAD+/NADH balance, promoting fatty acid synthesis and mitochondrial dysfunction, which contribute to fat accumulation and liver injury (16). In our EtOH-fed sham mice, reduced *Adh1* mRNA and *ADH1* proteins likely contributed to higher BAC and ALT, reflecting impaired EtOH metabolism. Conversely, OVX mice showed enhanced EtOH clearance and reduced toxic acetaldehyde accumulation *via* elevated *Aldh1a1* and *Adh1* mRNA levels. CYP2E1, key generator of ROS, was significantly elevated in EtOH-fed sham mice, correlating with increased LPO and ROS—hallmarks of oxidative stress (67). CYP2E1 overactivity depletes antioxidants like NQO1 and BHMT as shown in the EtOH-fed sham mice group, likely promoting ROS formation (68, 69), exacerbating oxidative stress, and impairing antioxidant defense. In addition, sham mice showed reduced *Sod2* mRNA levels, further indicating oxidative damage. Furthermore, we observed impaired methionine metabolism in the livers of both EtOH-fed sham and OVX mice, evidenced by lower SAM levels and reduced SAM/SAH ratios, markers associated with hallmark features of alcohol-related liver injury, including steatosis (70, 71, 72). Although no difference was detected between EtOH-fed sham and OVX mice in this regard, further studies are warranted to examine estrogen’s additional influence on methionine and the trans-sulfuration pathway. Also, *Ucp2* mRNA, a marker of oxidative mitochondrial ROS scavenger, was elevated in EtOH-fed sham mice, potentially as a feedback response to oxidative stress, consistent with observations in other liver injury models (73).

PXR and CAR are known as “sister receptors” for their shared role in hepatic lipid accumulation (74), with PXR expressed primarily in the liver and gut, and some reports in white adipose tissue (45). CAR activation, particularly, Cyp2b10 induction, is linked to hepatotoxicity, oxidative stress, steatosis, and impaired EtOH metabolism (35). Both PXR and CAR target genes, CYP3A11 and CYP2B10, are implicated in liver injury in alcohol use disorder (75). In this study, while EtOH ingestion had no significant effect on liver PXR and CAR mRNAs, it upregulated both nuclear receptors in pgWAT. Unexpectedly, both CYP3A11 and CYP2B10 mRNA and/or protein expression were significantly upregulated in livers and pgWAT of EtOH-fed sham mice, but marginally or absent in EtOH-fed OVX mice, suggesting the involvement of PXR–CAR activation in the hepatotoxicity we observed in the EtOH-fed sham mice. PXR–CAR may be driving hepatic steatosis in this model, with estrogen potentially enhancing PXR activation and thus exacerbating EtOH-induced liver damage (76). In addition to the inhibited expression of PXR–CAR targets, OVX mice had lower expression of ERα in the liver and adipose tissue than sham mice, indicating that EtOH likely enhances estrogen’s effects *via* Erα–PXR crosstalk. In EtOH-fed sham mice, hepatic *Erα* and PXR target gene expressions were increased, whereas NRF2—a critical antioxidant regulator—was inhibited, suggesting PXR, ERα, and NRF2 crosstalk contributes to hepatotoxicity. In contrast, OVX mice exhibited attenuated effects, possibly because of lower estrogen levels. PXR and NRF2 both play key roles in oxidative stress and xenobiotic metabolism (77, 78). Importantly, PXR deficiency protects against liver injury *via* NRF2/HO-1 pathway activation (79), and NRF2 inhibits lipid accumulation (80). Also, AhR activation potentially mediated by NRF2 (38) upregulates antioxidant gene *Nqo1*, conferring hepatoprotection (36, 81). AhR activation of NQO1 protects against oxidative damage by generating NAD+, which restores hepatic redox potential and supports oxidative metabolism (36). Also, AhR activation has been shown to modulate ERα and estradiol signaling by enhancing estrogen metabolism *via* the Cyp1a1–Cyp1a2 induction (82). Several studies in cancer biology have established a relationship between ERα-mediated estrogen signaling and the induction of Nrf2 (83, 84, 85). Notably, silencing ERα signaling has been associated with an upregulation of Nrf2 expression and its target genes (83, 84). Additional evidence highlights a complex interplay among AhR, ERα, and Nrf2 signaling pathways. For instance, ligand-activated AhR has been shown to reverse ERα-dependent repression of Nrf2 transactivation, thereby enhancing the expression of Nrf2 target genes such as NQO1 and HMOX1 (85). EtOH-fed OVX mice showed elevated AhR, NRF2, Cyp1a1, Cyp1a2, and NQO1, with unchanged BHMT protein, unlike sham mice where EtOH reduced the hepatic NQO1 and BHMT protein levels. This suggests that estrogen promotes EtOH-induced inhibition of protective proteins in sham mice, whereas OVX mitigates these effects.

Another important finding in this study is that EtOH-fed sham mice exhibited elevated hepatic EGR1, a transcription factor that enhances liver sensitivity to LPS and regulates genes involved in inflammation, fibrosis, and steatosis (34, 86, 87). This was accompanied by increased proinflammatory cytokines and chemokines, including *IL-6*, *IL-1β*, *TNF-α*, *Mip2*, and *Cxcl5*, indicating a strong hepatic inflammatory response. Supported by higher pgWAT IL-1β, TNF-α, CX3CL1, and MIP2 levels, which worsen liver injury (46). In contrast, OVX mice displayed a notable suppression of inflammatory markers, suggesting a protective effect of estrogen deficiency. Furthermore, serum cytokine analyses also revealed more elevated IL-6 and MCP-1 levels in EtOH-fed sham mice than in their OVX counterparts, indicating increased systemic inflammation in the presence of estrogen. However, the antiapoptosis protein, BCL-XL protein levels in the liver were elevated in EtOH-fed OVX mice but remained unchanged in the EtOH-fed sham mice, suggesting that estrogen signaling promotes apoptosis, enhancing hepatotoxicity. Together, the dampened inflammatory response and preserved antiapoptotic protein expression in EtOH-fed OVX mice point to estrogen's modulatory role in ALD.

The interplay between adipose tissue–derived hormones and liver injury in ALD was another focus of this current study. Interestingly, the roles of leptin in regulating hepatic lipid metabolism and inflammation under estrogen deficiency and chronic alcohol exposure and of adiponectin in balancing metabolic homeostasis yet associated with severe liver injury remains unclear. Future studies focusing on leptin and adiponectin signaling and their direct effects on hepatic injury in OVX and sham mice warrant further investigation. Since emerging evidence supports the clinical potential of NRF2 activation in combating oxidative stress and liver injury with omaveloxolone, a Food and Drug Administration–approved NRF2 agonist, showing efficacy in reducing oxidative damage and inflammation in other disease contexts (88), evaluating its hepatoprotective role against EtOH-induced hepatotoxicity in sham and OVX female mice in a chronic model of EtOH exposure will be astute. Understanding these interactions could provide insights into the metabolic and inflammatory crosstalk between adipose tissue and the liver plus exploring how PXR, ERα, NRF2, and AhR pathways interact with estrogen signaling will both provide critical insights into therapeutic approaches for ALD and related liver disorders.

In summary, our data indicate that estrogen exacerbates chronic EtOH–induced oxidative stress, inflammation, and hepatotoxicity in both liver and pgWAT. The observed reduction in EtOH-induced hepatotoxicity in OVX mice is likely because of downregulation of PXR–CAR target genes, *Cyp2b10* and *Cyp3a11*, which are associated with EtOH hepatotoxicity, and upregulation of *Adh1*/ADH1 and *Aldh1a1*, enhancing alcohol metabolism. Notably, our study demonstrates for the first time that OVX-induced estrogen reduction protects females against EtOH-induced liver damage *via* activation of the NRF2–HO-1 and AHR–NQO1 pathways, which may suppress oxidative stress and inflammation. These findings suggest that activation of NRF2–HO-1 and AHR–NQO1 signaling pathways could help prevent ALD in premenopausal females, whereas the damaging effects are mediated through a PXR–ERα–NRF2 crosstalk. Together, these results suggest the existence of an ERα–PXR–NRF2 signaling axis in liver and pgWAT, which contributes to sex differences seen in ALD, and demonstrates a previously unrecognized ERα signaling pathway, which contributes to sex differences in ALD.

## Experimental procedures

### Animal care, OVX, and sham surgeries, and alcohol treatment

Mice were matched by body weight and underwent either sham surgery or bilateral OVX. Buprenorphine (0.015 mg/ml) and carprofen (1 mg/ml) were administered subcutaneously at 0.1 mg/kg and 10 mg/kg, respectively, followed by anesthesia with xylazine/ketamine (80–100 mg/kg and 5–15 mg/kg, i.p) and continuous isoflurane (1%) during surgery. Through bilateral dorsal incisions, ovaries were accessed, externalized, and removed in the ovariectomized (OVX) group, whereas sham-operated mice retained intact ovaries. Muscle layers were sutured, and skin incisions closed with surgical clips, which were removed postrecovery. Recent reports have shown that the 10-day chronic EtOH feeding plus a single binge EtOH feeding (National Institute on Alcohol Abuse and Alcoholism [NIAAA] model) synergistically induces a more robust liver injury, inflammation, and steatosis as well as high blood alcohol levels than the Lieber–DeCarli liquid diet containing EtOH for 4 to 6 weeks (89). Furthermore, the NIAAA model has been found to mimic acute-on-chronic alcoholic liver injury in alcoholic patients suggesting its translational relevance (89). Therefore, we used the NIAAA model for our study. After a 3-week recovery period, sham and OVX mice were each divided into two groups (n = 8/group) and given free access to the Lieber–DeCarli control liquid diet ad libitum for 5 days to acclimatize them to the liquid diet. Following this, mice were fed either a 5% EtOH-containing liquid diet or pair-fed control diet for 10 days. On day 11, mice received a single gavage dose of EtOH (5 g/kg, 30% EtOH) or saline and were euthanized 9 h later under isoflurane anesthesia. Blood samples were collected *via* cardiac puncture, and liver and pgWAT tissues were harvested, weighed, and stored at −80 °C. The diets, based on the Lieber–DeCarli EtOH formula from DYETS, Inc, provided 1 kcal/ml with 18.9% protein, 16.5% fat, and 64.5% carbohydrates. All procedures were approved by the University of Tennessee Health Science Center (UTHSC) Institutional Animal Care and Use Committee, in compliance with the National Institutes of Health's guidelines for the care and use of laboratory animals.

### Serum biochemical analysis

Blood was centrifuged at 8000 rpm for 20 min at 4 °C to obtain serum and immediately frozen at −80 °C for BAC, liver enzyme assay, including ALT, triglycerides, NEFAs, cholesterol, cytokine, and bile acid analysis. The L3k assay kit (Sekisui Diagnostics P.E.I., Inc) was used to quantitatively measure EtOH concentration per the manufacturer’s protocol, whereas bile acid was measured using a commercially available kit (Diazyme Laboratories, Inc). Serum cytokine and chemokine levels were analyzed using the Cytokine/Chemokine 32-Plex Discovery Assay Array (MD32) by Eve Technologies. For liver function, an ALT activity kit from MilliporeSigma was used to measure serum ALT levels.

### Serum and hepatic lipids (triglycerides, NEFA-HR, and cholesterol), total hepatic GSH, ROS, SAM, SAH, and adenosine measurement

Total hepatic lipids were isolated from 100 mg of liver tissue homogenate using the freshly prepared chloroform:methanol (2:1) extraction method described previously (90). Serum and hepatic lipids were analyzed spectrophotometrically per the manufacturer’s protocols using commercial assays kits (Wako Pure Chemical Industries). Fresh liver tissue was used for the determination of hepatic SAM and SAH. Briefly, 50 mg of fresh liver tissue was homogenized in 400 μl of 0.5 N perchloric acid. Following centrifugations at 20,000*g*, the perchloric acid extract was subjected to HPLC as previously described (71, 91). Total GSH levels were measured with a commercially available kit per the protocol provided by Cayman Chemicals (catalog no.: 703002). Hepatic ROS levels were assessed using the 2,7-dichlorodihydrofluorescein diacetate method, as previously described (92). The list of reagents, assays, source, and catalog numbers used are shown in Table 1.

**Table 1:** List of reagents and sources

Commercial assays	Source	Catalog number
BCA protein Assay	ThermoFisher	23225
ALT Activity Assay	Sigma–Aldrich	MAK052
TBARS Assay Kit	Zeptometrix	0801192
Free Cholesterol E	Fujifilm	99302501
L-Type Triglyceride M family product	Fujifilm	994–02891
NEFA HR (2) family product	Fujifilm	999–34691
Superscript VILO cDNA synthesis kit	ThermoFisher	11755-500
TRIzol (Triazole)	Ambion Life Technologies	15596018
TaqMan Universal PCR Master Mix	ThermoFisher	444557
Power SYBR Green PCR Master Mix	ThermoFisher	4367659
Intercept TBS blocking buffer	Li-Cor	927-60001
Intercept T20 (TBS) antibody diluent	Li-Cor	927-65001
Revert 700 Total Protein Stain	Li-Cor	827-15733
Image Studio Lite	Li-Cor	Version: 5.2
GraphPad	Dotmatics	Version: 10.0
Lieber/DeCarli Regular Liquid Diet for Rodents	Dyte, Inc	Control (710027); Ethanol (710260)
BAC Assay Kit (L3K)	Sekisui Diagnostics LLC/ThermoFisher	273-30/NC9764936
Bile Acid Assay Kit	Diazyme	D2042A-KO1
Glutathione Assay Kit	Cayman Chemical	703002

### Liver lipid peroxidation

Liver LPO was assayed using the TBARS Kit (ZeptoMetrix). Specifically, approximately 100 mg of liver tissues were homogenized in 2 ml of PBS. Sample homogenates (100 μl), as well as malondialdehyde standards, were incubated with SDS and 0.8% thiobarbituric acid (20% acetic acid, pH 3.5) in the presence of 0.8% butylated hydroxytoluene at 95 °C for 1 h. After incubation, samples were cooled on ice and centrifuged at 3000 rpm for 15 min. Supernatants were read on a spectrophotometer at 532 nm. Protein contents in liver homogenates were determined by the Bicinchoninic Acid Protein Assay protein assay kit (Thermo Scientific). TBARSs were expressed as micromoles of malondialdehyde equivalents per gram of liver.

### H&E staining and Oil Red-O staining

Histological examination of the liver was performed by either fixing liver samples in 10% formalin for H&E staining or snap freezing liver samples in liquid nitrogen for Oil Red-O staining. Samples were then embedded and stained with H&E stain or Oil Red-O stain for histological feature analysis. Images were captured using an Olympus IX73 microscope and cellSens Standard Software (Olympus) at a magnification of 60×. In a blind liver H&E histology examination, an independent pathologist graded the percentage of liver cells containing lipids as follows: <5% = 0, 5% to 33% = 1, 33% to 36% = 2, >66% = 3 with lobular inflammation rated as follows 0 (<1 foci/200×); 1 (<2 foci/200×); 2 (2–4 foci/200×); and 3 (>4 foci/200×) as previously described (93).

### Quantification of liver and pgWAT mRNA levels using real-time quantitative PCR

Liver and pgWAT mRNA levels were extracted using the TRIzol reagent (Ambion Life Technologies-Invitrogen) per the manufacturer’s instructions. mRNA concentrations were determined using the NanoDrop Spectrophotometer Technology (ThermoFisher Scientific) and standardized at a concentration of 1 ng/ml for the synthesis of first-strand complementary DNA using an oligo(dT) primer and Superscript Vilo IV Reverse Transcriptase (ThermoFisher Scientific). Real-time quantitative PCRs were carried out with specific SYBR Green and TaqMan primers (Applied Biosystems) on a Quant-studio 6 Pro Real-Time PCR System (Applied Biosystems) as previously described (34). The SYBR Green primer sequences used are shown in Table 2, which have been previously published (34). Furthermore, the list of proprietary TaqMan Gene Expression Assays used for real-time quantitative PCR were purchased from Applied Biosystems/Life Technologies, which is shown in Table 3.

**Table 2:** List of primer sequence, 5′-3′ (SYBR Green)

Gene	Forward	Reverse
Bhmt	AAGCCTTAAAAGCATCTGGTAAGC	GGCACGCCATGCAGATC
Tnfα	TTTCTCCTGGTATGAGATAGCAAATC	ACAAGGCTGCCCCGACTAC
Mip2	CCCTTGAGAGTGGCTATGACTTC	GAACATCCAGAGCTTGAGTGTGA
Nf-ϏB	TGGCAGCTCTTCTCAAAGCA	CCAAGAGTCGTCCAGGTCATAGA
Ccr2	AGCACATGTGGTGAATCCAA	TGCCATCATAAAGGAGCCA
Fxr	CAC GAA GAT CAG ATT GCT TTG C	CCGCCGAAC GAAGAA ACA T
Cyp7a1	TGACCCAGACAGCGCTCTTT	CCATGATGCAAAACCTCCAAT
Shp	GTCTTCAAGGAGTTCAGTGATGTCA	CAGGCACCCTTCTGGTAGATCT
Mrp2	TTT GAT CCT TTC AGC CTT TTC AG	CGTAACGCTACTCAGAAATGAAGC T
Bsep	CAG AAC ATG ACA AAC GGA ACA AG	CCT GCG TAG ATG CCA GAA AAT T
Cyp8b1	TCGCACACATGGCTCGAT	GCCCTTACTCCAAATCCTACCA
Rxr	CATGAGTTAGTCGCAGACATGGA	CGACCCGTTGGAGAGTTG AG
Mttp	CCGCTGTGCTTGCAGAAGA	TTTGACACTATTTTTCCTGCTATGGT
Cyp3a11	CCCGCCGGTTTGTGAAG	TCACAGACCCAGAGACGATTAAGA
Pparα	TGCTTTTTCAGATCTTGGCATTC	GATTCAGAAGAAGAACCGGAACA
Cpt1α	GCCGTGCTCTGCAAACATC	CGATCATCATGACTATGCGCTACT
Cyp4a14	GGTGTTGGCAAGGCATTCC	CAAGACCCTCCAGCATTTCC
Acc1α	TGCTCCGCACAGATTCTTCA	ATGTCCGCACTGACTGTAACCA
Fas	GGACCGAGTAATGCCATTCAG	CCCGGAGTCGCTTGAGTATATT
Scd1	CGT TCC AGA ATG ACG TGT ACG A	AGG GTC GGC GTG TGT TTC
Scd2	TTTTCTTTGCTTTGTCCCTGATG	TGGTGTACTCTGGAAGGTGAACA
CD36	TGGAGATTACTTTTTCAGTGCAGA A	TCCAGCCAATGCCTTTGC
PparƔ	CCC AAT GGT TGC TGA TTA CAA A	GAGGGAGTTAGAAGGTTCTTCATGA
Srebp1-c	TGTTGCCATGGAGATAGCATCT	CATGCCATGGGCAAGTACAC
Cyp2b10	TTCTGCCCTTCTCAACAGGAA	ATGGACGTGAAGAAAAGGAACAAC
Gapdh	CCTGCTTCACCACCTTCTTGA	TGTGTCCGTCGTGGATCTGA

**Table 3:** List of primer (TaqMan)

TaqMan primer (mouse)	Source	Identifier
Adh	ThermoFisher	Mm00507711_m1
Adh4	ThermoFisher	Mm00478838_m1
Adiponectin	ThermoFisher	Mm00456425_m1
AHR	ThermoFisher	Mm00478932_m1
Akr1b8	ThermoFisher	Mm00484314_m1
Aldh1a1	ThermoFisher	Mm00657317_m1
Aldh2	ThermoFisher	Mm00477463_m1
Bcl2	ThermoFisher	Mm00477631_m1
Car	ThermoFisher	Mm01283978_m1
Catalase	ThermoFisher	Mm00437992_m1
Fsp27/Cidec	ThermoFisher	Mm00617672_m1
Cx3cl1	ThermoFisher	Mm00436454_m1
Cxcl5	ThermoFisher	Mm00436451_g1
Cyp1a1	ThermoFisher	Mm00487218_m1
Cyp1a2	ThermoFisher	Mm00487224_m1
Cyp2a5	ThermoFisher	Mm00487248_m1
ERα	ThermoFisher	Mm00433149_m1
ERβ	ThermoFisher	Mm00599821_m1
Fgf21	ThermoFisher	Mm00840165_g1
Gper1	ThermoFisher	Mm02620446_s1
Gstm3	ThermoFisher	Mm00833923_m1
Hmox1	ThermoFisher	Mm00516005_m1
Il-1β	ThermoFisher	Mm00434228_m1
Il-6	ThermoFisher	Mm04934123_m1
Nqo1	ThermoFisher	Mm01253561_m1
Nrf2	ThermoFisher	Mm00477786_m1
Pxr	ThermoFisher	Mm01344139_m1
Sod2	ThermoFisher	Mm01313000_m1
Ucp2	ThermoFisher	Mm00627599_m1
Gapdh	ThermoFisher	Mm99999915_g1
Leptin	ThermoFisher	Mm00434759_m1

### Preparation of liver homogenate, microsomal, and cytosolic fractions for Western blot

Approximately, 100 mg frozen liver samples were homogenized in lysis buffer (10 mM Hepes, pH 7.9, 100 mM KCl, 1.5 mM MgCl_2_, 0.1 mM EGTA, 0.5 mM dithiothreitol, 0.5% Nonidet P-40, and protease inhibitor cocktail) at 4 °C and centrifuged at 10,000*g* for 30 min. The supernatant was collected as the cytosolic fraction. Microsomal fractions were separated from fresh liver tissue as described previously (94). Cytosolic fractions (30 μg/lane) were mixed with Laemmli loading buffer containing β-mercaptoethanol, boiled for 5 min, and subjected to Western blot analysis as previously described (34). For liver homogenate preparation, about 100 mg of frozen liver tissues were obtained and homogenized on ice for Western blot analysis as previously described (34). The protein concentrations for the liver homogenate as well as the microsomal and cytosolic fractions were determined using the Bicinchoninic Acid Protein Assay kit (ThermoFisher Scientific). All samples were diluted to obtain a final protein concentration of 5 μg/μl. The different liver fractions were mixed in Laemmli loading buffer containing β-mercaptoethanol, boiled for 5 min, and separated (20–30 μg/lane), on a 10% or 15% SDS-PAGE gels, and transferred to polyvinylidene difluoride membrane. Membranes were blocked in LI-COR Intercept Blocking Buffer and probed with primary antibodies diluted in antibody dilution buffer, overnight. The list of primary and secondary antibodies, source, catalog numbers, and dilutions used are shown in Table 4. The next day, membranes were washed with 20% Tween in Tris-buffered saline, followed by incubation with IRDye-conjugated secondary antibodies in antibody dilution buffer, and immunoglobulins were visualized using the LI-COR detection system. Quantitation was performed using the Image Studio Lite Version 5.2 software (LI-COR Biosciences).

**Table 4:** List of antibodies

Primary antibody	Source	Catalog number	Dilution
FGF21	Invitrogen	MA532652	1:1000
ADH	Santa Cruz	Sc-137078	1:200
ALDH2	Santa Cruz	Sc-48837	1:1000
ALDH1B1	Santa Cruz	Sc-393583	1:1000
CYP2E1	Abcam	AB28146	1:1000
LFABP1	Santa Cruz	Sc-374537	1:200
CPT1α	Abcam	AB128568	1:1000
CYP4A14	Santa Cruz	Sc-271983	1:500
PPARƔ	Santa Cruz	Sc-7273	1:1000
CIDEC (FSP27)	Abcam	AB198204	1:1000
EGR1	Cell Signaling	4153S	1:1000
BCL-XL	Cell Signaling	2764S	1:1000
FAS	Cell Signaling	3180S	1:1000
ACC1A	Cell Signaling	3676S	1:1000
CYP3A	Santa Cruz	Sc-271033	1:500
CYP2B10	Abcam	AB9916	1:500
NQO1	Abcam	AB34173	1:1000
BHMT	Abcam	AB96415	1:1000
IRDye 800CW Goat anti-Rabbit IgG Secondary Antibody	Li-Cor	926-32211	1:5000
IRDye 800CW Goat anti-Mouse IgG Secondary Antibody	Li-Cor	926-68070	1:5000
IRDye 800CW Donkey anti-Goat IgG Secondary Antibody	Li-Cor	926-32214	1:5000
α-tubulin	Cell Signaling	2144S	1:1000

### Statistical analyses

All results were presented as means ± SEM. Statistical analyses were performed using GraphPad Prism, version 9.0 for Windows (GraphPad Software, Inc). Differences between groups were analyzed using two-way ANOVA followed by Tukey multiple comparisons test. A *p* value of <0.05 was considered statistically significant.

### Ethical consideration

Female C57BL/6N Taconic mice (2–3 months old) generated from breeding pairs obtained from Taconic Biosciences were used and cared for per the UTHSC’s Institutional Animal Care and Use Committee. Mice were housed in standardized polycarbonate cages on racks directly vented *via* the facility's exhaust system at 22 °C with a 12-h/12-h/dark cycle. Animals received humane care, and all procedures were conducted in accordance with the Declaration of Helsinki and National Institutes of Health Guidelines for the Care and Use of Laboratory Animals and were approved by the UTHSC Institutional Animal Care and Use Committee.

### Data availability

The datasets supporting the conclusions of this article are included within the article and its additional files.

## Conflict of interest

The authors declare that they have no conflicts of interest with the contents of this article.
